# Edible Plant Extracts against *Aedes aegypti* and Validation of a *Piper nigrum* L. Ethanolic Extract as a Natural Insecticide

**DOI:** 10.3390/molecules28031264

**Published:** 2023-01-28

**Authors:** Lais Silva Morais, João Paulo Barreto Sousa, Carolina Melo Aguiar, Ciro Martins Gomes, Daniel Pecoraro Demarque, Lorena Carneiro Albernaz, Laila Salmen Espindola

**Affiliations:** 1 Laboratório de Farmacognosia, Universidade de Brasília, Campus Universitário Darcy Ribeiro, Asa Norte, Brasília CEP 70910-900, DF, Brazil; 2 Programa de Pós-Graduação em Ciências Médicas, Faculdade de Medicina, Universidade de Brasília, Campus Universitário Darcy Ribeiro, Asa Norte, Brasília CEP 70910-900, DF, Brazil; 3 Laboratório de Farmacognosia, Faculdade de Ciências Farmacêuticas, Universidade de São Paulo, Av. Professor Lineu Prestes, 580, São Paulo CEP 05508-900, SP, Brazil

**Keywords:** *Aedes aegypti*, *Piper nigrum* L., black pepper, accelerated solvent extraction (ASE), standardized extract, validation

## Abstract

The *Aedes aegypti* mosquito significantly impacts public health, with vector control remaining the most efficient means of reducing the number of arboviral disease cases. This study screened the larvicidal and pupicidal activity of common edible plant extracts. *Piper nigrum* L. (black pepper) extract production was optimized using accelerated solvent extraction (ASE) and validated following regulatory requirements using HPLC-PDA analytical methodology to quantify its major component–piperine. Larvicidal activity was determined for the standardized *P. nigrum* fruit ethanol extract (LC_50_ 1.1 µg/mL) and piperine standard (LC_50_ 19.0 µg/mL). Furthermore, 9-day residual activity was determined for the extract (4 µg/mL) and piperine (60 µg/mL), with daily piperine quantification. Semi-field trials of solid extract formulations demonstrated 24-day activity against *Ae. aegypti* larvae. Thus, the standardized *P. nigrum* extract emerges as a potential candidate for insecticide development to control the arboviral vector.

## 1. Introduction

Arboviral diseases, including dengue, Zika, chikungunya and urban yellow fever continue to pose a significant health concern worldwide, with a vaccine available for the latter only [1]. *Aedes aegypti* is a notably adaptable vector capable of acquiring resistance to the principal chemical control agents [2]. Therefore, the need remains to source new insecticides to reduce insect numbers targeting all life cycle stages, particularly at aquatic area breeding sites during the combined larvae/pupae development period (7–14 days) [3].

Natural products constitute a source of compounds with insecticidal properties [4]. Numerous studies have reported the activity of edible plant extracts and essential oils against *Ae. aegypti*, highlighting readily available matrices [5]. However, standardization extraction and regulatory criteria continue to pose a significant challenge to obtaining a final product [6].

Method validation is an essential quality assurance process, as it ensures accurate reproduction of a reliable product. This process focuses on specifying sample characteristics, quantifying chemical constituents and permitting quality control. This process is more complex for natural products considering the number of metabolites involved, perhaps accounting for the lack of approved natural insecticides. One example is neem oil (*Azadiractha indica* A. Juss.), a natural insecticide approved in Brazil, the European Union and the United States for *Ae. aegypti* control [7,8].

**Figure 1:**
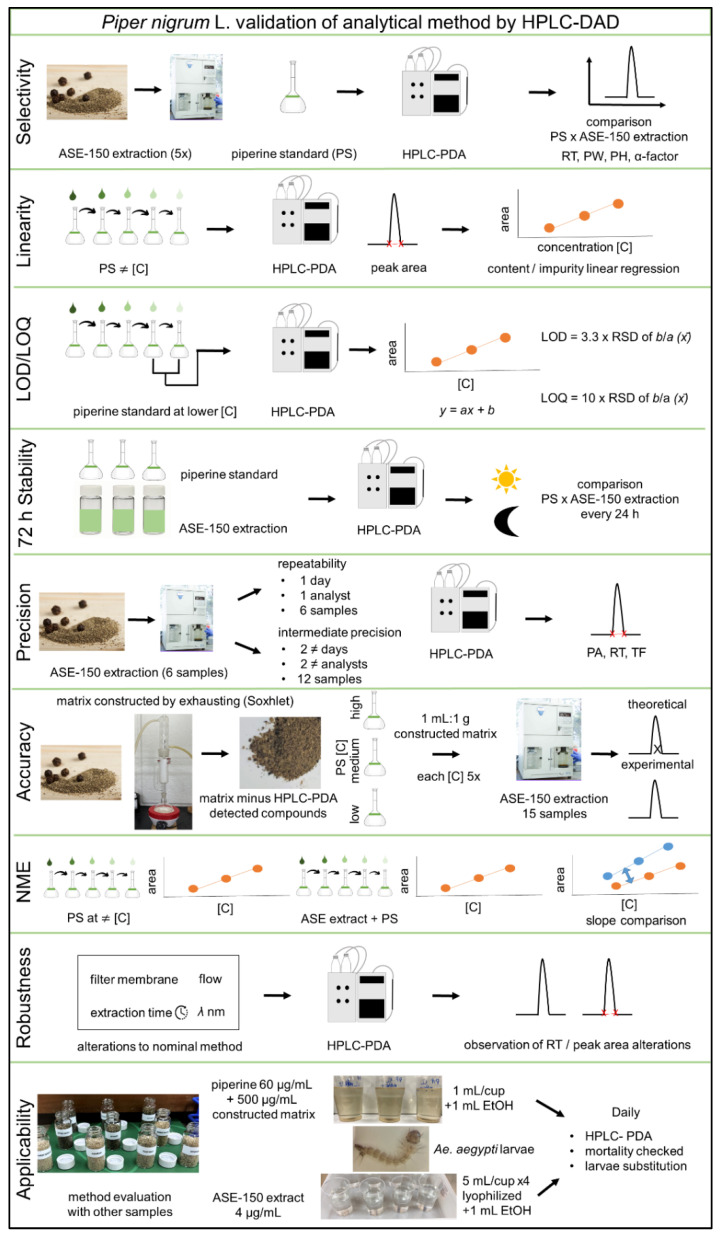
Overview of the steps involved in validating the *Piper nigrum* fruit (black pepper) ethanolic extract analytical method and its application in *Ae. aegypti* larvicidal assays.

Another plant species with documented larvicidal activity against *Ae. aegypti* is *Piper nigrum* L. (black pepper, Piperaceae), rich in piperamides, particularly piperine, responsible for its characteristic flavor and biological activities [9,10]. Despite a number of reports relating to *Piper* spp. formulations and activity against different stages of *Aedes* spp., comprehensive studies, including field trials and quantification, remain necessary to address this gap in the literature [6,11,12] and obtain a larvicidal product. Therefore, the aims of this study were to: (i) screen 70 different edible plant extracts for larvicidal/pupicidal activity against *Ae. aegypti*; (ii) produce a standardized, validated *P. nigrum* ethanolic extract with parallel piperine quantification (Figure 1); (iii) develop a solid formulation; and (iv) perform simulated small-scale field trials.

## 2. Results and Discussion

### 2.1. Screening of Edible Plant Extracts against Ae. aegypti Larvae and Pupae

A total of 70 edible plant extracts were produced by accelerated solvent extraction (ASE 150^®^) using a mixture of different polarity solvents—hexane:dichloromethane:ethyl acetate:ethanol (4:1:4:1), a strategy enabling the extraction of compounds with different polarities while maintaining the major nonpolar proportion, reported as most active [6]. The ASE technique is a more efficient extraction process, requiring less solvent and time, allowing the control of some of the test parameters. These characteristics contribute to the evaluation of screening samples [13]. Matrices were selected to provide a representative sample of fruits and vegetables, medicinal plants commonly used as tea, and spices readily available in local markets. The larvicidal and pupicidal activities of the edible plant extracts, together with their respective yields, were determined (Table 1). Fifteen samples caused 100% L3 larvae mortality: Florida burrhead (3), chamomile (9), pitaya (18), blueberry (23), avocado peel (33), avocado pulp (34), Hass avocado seeds (37), sassafras (39), black pepper (51), goji berry (56), chili pepper (58), yellow scorpion pepper (60), cumari pepper (63), white raisin (68) and cardamom (69), while only five caused 100% pupae mortality: yacon (15), *olho de boi* (27), avocado pulp (34), cocoa powdered seed (43), and chili pepper (58). Although pupae are more resistant, three extracts caused significant mortality at both stages—avocado pulp (34) and chili pepper (58) caused 100% larvae and pupae mortality, while yellow scorpion pepper (60) caused 100% larvae and 94% pupae mortality. As larvae filter water, they are more exposed to chemical control compounds than pupae which do not feed and require surface contact [14]. Some of the edible plants tested herein have previously reported activity against *Ae. aegypti*: cardamom aqueous extract (LC_50_ 43.58 µg/mL) and chamomile essential oil (LC_50_ 2.9 to 60.5 µg/mL, depending on origin) [15,16]. Considering the data obtained, *Piper nigrum* was chosen for extraction optimization due to its larvicidal activity, sample yield and ready global availability, rendering it the most feasible for future large-scale production.

### 2.2. Extraction Optimization

**Figure 2:**
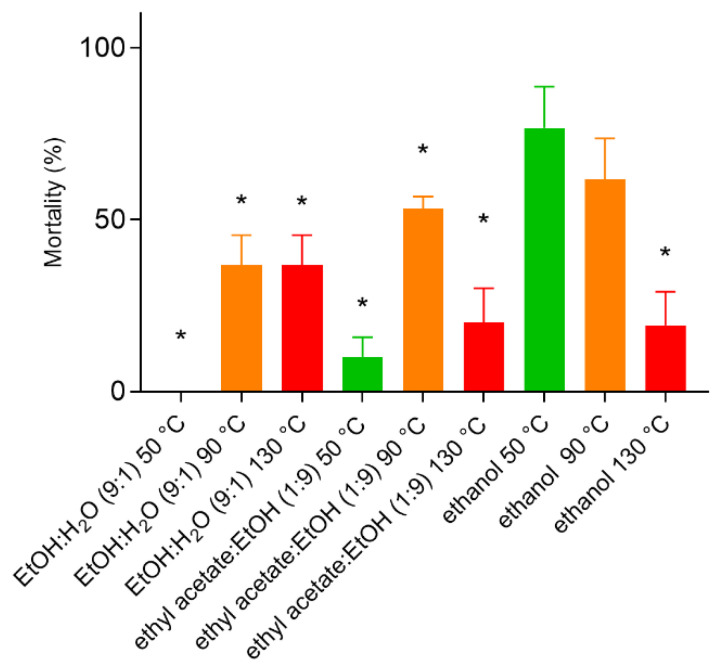
*Aedes aegypti* L3 larvae mortality after 24 h exposure to different *Piper nigrum* extracts (1.9 µg/mL). EtOH: ethanol; H2O: distilled water. * Significant statistical difference (*p* < 0.05) when compared to 50 °C-prepared ethanolic extract by one-way ANOVA and Dunnett’s post hoc test.

Different variables—“green solvents” [17] and temperature—were studied to optimize extraction, involving a total of nine extractions using: ethanol:water (9:1), ethyl acetate:ethanol (1:9) and ethanol at three temperatures: (50, 90 and 130 °C). These extracts were tested at 1.9 µg/mL against *Ae. aegypti* L3 larvae, with percentage mortality determined after 24 h. These solvents were used to investigate potentially enhanced activity by increasing/reducing polarity. The ethanol extract demonstrated better activity at 50 and 90 °C, while the 130 °C ethanol:water (9:1) achieved the highest larvae mortality. ASE has an inert N_2_ atmosphere that enables extractions in higher temperatures [18]; however, considering extract stability and industrial safety requirements, the lower temperature (50 °C) ethanol constituted the best solvent option. Mortality levels varied according to extraction temperature, probably due to differences in the *P. nigrum* extract chemical profiles. The 50 °C ethanolic extract was the most active (Figure 2), and thus selected for optimization as a fixed parameter.

**Figure 3:**
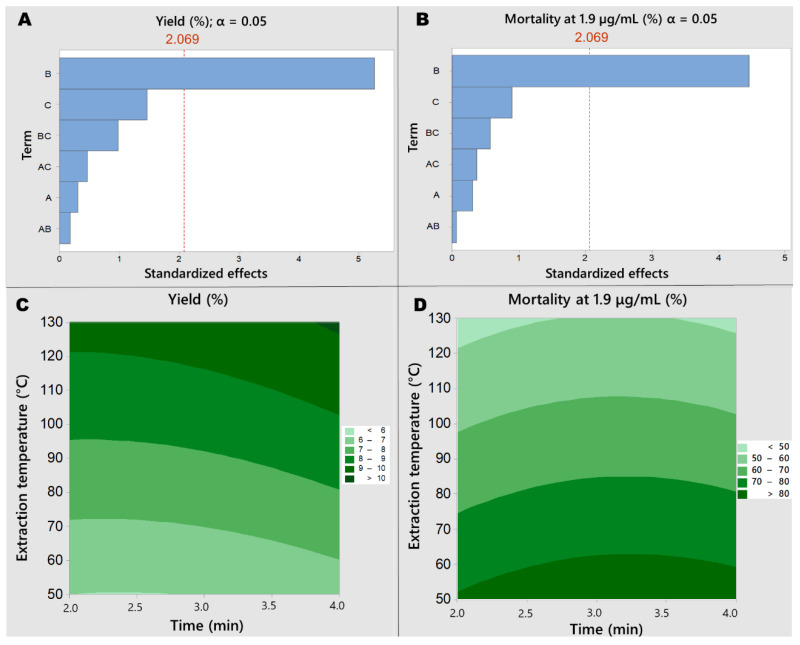
Pareto charts and contour scales of the responses applying Box–Behnken design to *Piper nigrum* extractions. (**A**): Pareto chart for yield. (**B**): Pareto chart for *Aedes aegypti* larvae mortality (1.9 μg/mL, 24 h). y-axis Term: A-time; B-temperature; C-sample quantity. Broken red line indicates statistically significant effects (Student’s *t*-test, 95% confidence level). (**C**): contour scale for yield. (**D**): contour scale for *Aedes aegypti* L3 larvae mortality (1.9 μg/mL, 24 h). Sample quantity: 3 g.

After variable determination (solvent and temperature), the factorial design experiments were executed, with yield and activity against *Aedes aegypti* L3 larvae (1.9 µg/mL) recorded (Table S1, Supplementary Material). A total of 15 extractions (in duplicate) were conducted varying: temperature, sample quantity and static cycle duration. The resulting extracts were tested at 2.5, 2.0 and 1.9 μg/mL. The 1.9 µg/mL concentration was selected as it had the capacity to cause different mortality between the extractions, but still had samples that killed 100% larvae. In factorial design, different responses to variations allowed parameters to be studied. Subsequent Pareto and contour scales were plotted, with the Pareto analysis showing that temperature was the only statistically significant variable for both responses (Figure 3A,B). However, the contour plot generated showed a negative relationship: as temperature increases, so did the yield, but activity decreased. Considering that the *P. nigrum* extract yields varied from 4 to 13%, we selected the most active extraction temperature (50 °C) (Figure 3C,D). To analyze samples with minimum manipulation, we also specified 1 g as the sample quantity, enabling sample preparation directly from the ASE-150 extractive solution. The static cycle was set at 4 min.

A previous study reported that higher temperatures in ASE extractions increased yields of *P. nigrum* extracts from different origins using different solvents, [19] as observed in the present study. The authors also highlighted the drawbacks of other extraction methodologies, such as supercritical fluid or microwave, including cost and equipment preparation time. This study affirms that the optimized extraction process, guided by biological activity tests, can result in an extract active at low concentrations (*Ae. aegypti* larvae, 1.9 μg/mL). A conventional ethanolic extraction of *P. nigrum* by maceration was reported by Souza [20], involving three 72-h maceration cycles, yielding 6.4%, similar to the average ASE yield (6.3%) obtained herein at 50 °C. Not only did ASE extraction take a fraction of the time (approximately 20 min), but it also employed considerably less solvent (175 mL), highlighting the advantages of this method over traditional techniques. To validate our extraction method, we used piperine, a major compound in *P. nigrum*.

### 2.3. Optimized P. nigrum Extract: Analytical Method Development and Validation

**Figure 4:**
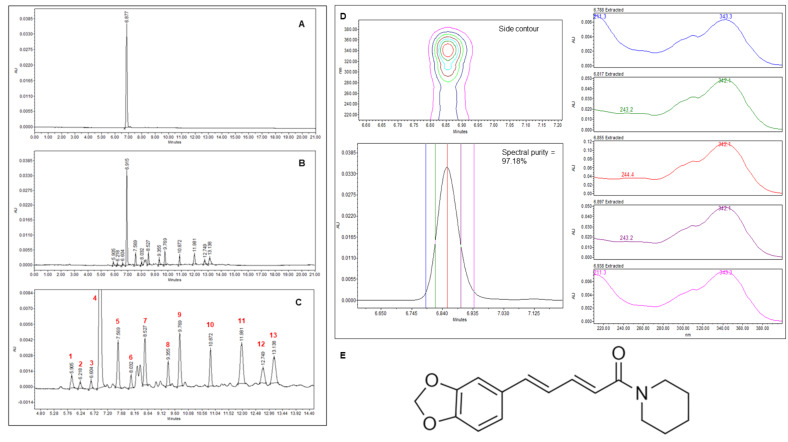
Validated method selectivity. (**A**). Piperine chromatographic profile (266 nm). (**B**). Chromatographic profile of the standardized *Piper nigrum* ethanolic fruit extract (266 nm). (**C**). B amplified. (**D**). Spectral purity calculation for piperine standard, isolated using the average of 5 spectral peak data. (**E**). Piperine molecular structure.

Analytical methodology development involved evaluating different conditions: stationary/mobile phases, elution gradient, PDA-UV detector wavelength and filter membranes to obtain the best chromatographic profile for the optimized extract (Section 3.1). Selectivity was obtained by comparing the standard piperine solution profile (Figure 4A) with the extract (Figure 4B,C), targeting adequate separation of the major compound (piperine) from the other peaks. Thirteen peaks, including piperine, were monitored considering: retention time, α factor, peak height and width. We were able to observe adequate selectivity, whereby the standard deviation for all chromatographic variables was <5%, with piperine spectral purity determined using the average of 5 UV spectral peak data (Figure 4D). All of the raw data for selectivity is presented in Table S2.

Peak 4 was identified as piperine by retention time and peak spectral purity verification using the external standard. The isolated piperine ^1^H NMR data is also reported (Supplementary Material Figure S12). Minor peaks were monitored during the validation process; however, since they were not available for complete validation, only piperine identification was conducted.

Linearity was determined in two levels according to concentration: content, ranging from 11.5 to 263.6 µg/mL, and impurity, from 1.5 to 48.5 µg/mL (raw data—Tables S3 and S4). The peak area and concentration correlation curves, expressed by R2, were >0.99 for both datasets. The linear regression equations for content and impurity were y =1069.4x − 2010 and y = 1115.3x − 421.72, respectively (Figures S1 and S2). The residue study data is detailed in Tables S5 and S6. Figures S3 and S4 confirmed that the models presented adequate piperine quantification at different points, with maximum standardized residue ±2.5%. The curve statistics are described in Tables S7 and S8. Detection and quantification limits were 0.51 and 1.54 μg/mL, respectively, confirmed by experimental analysis (Figure S5). The RSD value at the quantification level (4.4%) was calculated as the average of RSD values from the slope and y-intercept of three independent calibration curves. Some methods in the literature have lower limits; however, in order to better observe minority compounds, we selected the 266 nm wavelength, resulting in higher piperine detection and quantification limits, given that its maximum absorbance is 344 nm [21]. A sample stability study was conducted, in which the relative content of 13 peaks were monitored, including piperine in the standardized extract (from ASE-150) and piperine only in ethanol. An 18.94% reduction was observed in the piperine standard solution (in ethanol) at 72 h, while in the ASE-extract, it was only 5.97% (72 h). No significant reduction was observed for the other peaks, with relative content comparable to the initial analysis (98%). Preliminary sample stability data provided information about how the standardized extract must be manipulated (Tables S9 and S10; Figures S6 and S7). Method precision was evaluated by repeatability and intermediate precision, analyzing variations in the retention time, peak area and tail factor for the 13 peaks studied. The RSD for the variables were monitored with the highest value (6.1%) demonstrating acceptable method precision (Tables S11 and S12). Accuracy was determined by recovery, comparing the theoretical piperine concentration to the experimental value at three levels, as described in the methodology (Section 3.5). The different level recovery percentages: 92.73 (high), 88.88 (medium) and 83.36 (low) validated the method accuracy. The RSD of the percentages obtained were 5.10, 4.76 and 5.70, respectively. The maximum error obtained for the low level was 16.64% (Table S13). The ASE-150 extraction and sample preparation procedures, including filtration, showed adequate method accuracy. The normalized matrix effect (NME) was calculated by constructing calibration curves: piperine standard (PS), a standardized extraction enriched with the piperine standard (SE + PS), and a theoretical curve constructed from the latter excluding the initial piperine concentration (IPC) present in the standardized extraction (SE + PS − IPC). The ratio of the slopes compared to the PS curve were 0.97 and 0.96 for SE + PS and SE + PS − IPC, respectively. These ratios, proximal to 1.0, showed that the curves are similar, thus supporting the absence of the matrix effect (Figure S8 and Table S14). Robustness was assessed by evaluating ASE-150 extraction time, filter membrane, wavelength and flow rate (Table 2) to identify which of these parameters altered piperine retention time (RT) or peak area. The flow rate was the only parameter that exceeded 10% effect for both RT and peak area, altering the piperine chromatographic profile and, as such, should be controlled (Figure S9).

This information, in conjunction with the extraction, sampling and HPLC parameters, confirms method robustness (Tables S1–S14 and Figures S1–S9). To demonstrate method applicability, 10 different samples of commercially acquired black (5) and white (5) pepper were submitted to standardized extraction and the validated method to determine piperine levels. The black pepper berry differs from the mature white pepper berry in that it does not possess peel and involves different post-collection procedures [22]. The method quantified piperine in the 10 different *Piper nigrum* commercial samples, with content ranging from 1.6 to 2.5%. Thus, the method is appropriate for raw material quality control (Table S15). Extract piperine concentration was monitored during validation. The ASE *P. nigrum* ethanolic extract validated herein reached 46.7 ±1.7% piperine, corresponding to 28 mg/g of the amide in powdered black pepper. Piperine content in the literature varies from 2 to 9% [23], while our study determined 2.8%. Some characteristics, including the technique for determination and distinct types of extractions, are responsible for different concentrations not excluding cultivation parameters. Compared to other extraction techniques, including supercritical fluid, ultrasound maceration and Soxhlet, ASE is one of the least time-consuming (15 min) and does not require filtration steps/cooling time prior to handling [24].

### 2.4. Larvicidal Activity against Aedes aegypti

The standardized validated extract and piperine standard were submitted to larvicidal assays to determine the LC_50_ and LC_90_ values (Table 3, Figures S10 and S11). After 24 h, piperine values were LC_50_ 19.0 μg/mL and LC_90_ 38.1 μg/mL, while the standardized extract values were LC_50_ 1.1 μg/mL and LC_90_ 1.8 μg/mL. Piperine, the major compound in *P. nigrum*, tested herein demonstrated potential when compared to other compounds/extracts in the literature (LC_50_ < 50 μg/mL) [6]. In addition, its elevated concentration in the extract contributes to the larvicidal activity. Piperine has an important role in biosynthesis as a source of different isomers, including other alkamide production, conferring organoleptic and other biological activity [20,21,22]. Other authors reported the activity of different alkamides with lower LC_50_ (0.04 μg/mL) [10] values even when compared to the standardized extract produced herein (LC_50_ 1.1 μg/mL). Therefore, a *P. nigrum* sourced standardized extract containing piperine and minor compounds (including other alkamides), produced using a green solvent and a more efficient process than alkamide isolation, constitutes an innovative natural insecticide. This activity is conferred by the presence of unsaturated aliphatic chains, with methylenedioxy and amide groups in the different alkamide compounds [19,25]. The potent activity of the validated *P. nigrum* ethanolic extract certainly results from the synergy of piperine (47%) together with minor compounds. This major compound is crucial for larvae mortality since its high relative content does not reduce extract potency. In addition, some of the minor compounds may possess important individual activity [10], supporting direct use of the extract in new insecticide development, not to mention streamlining production.

### 2.5. Applicability of Validated Method: Residual Larvicidal Activity against Aedes aegypti

**Figure 5:**
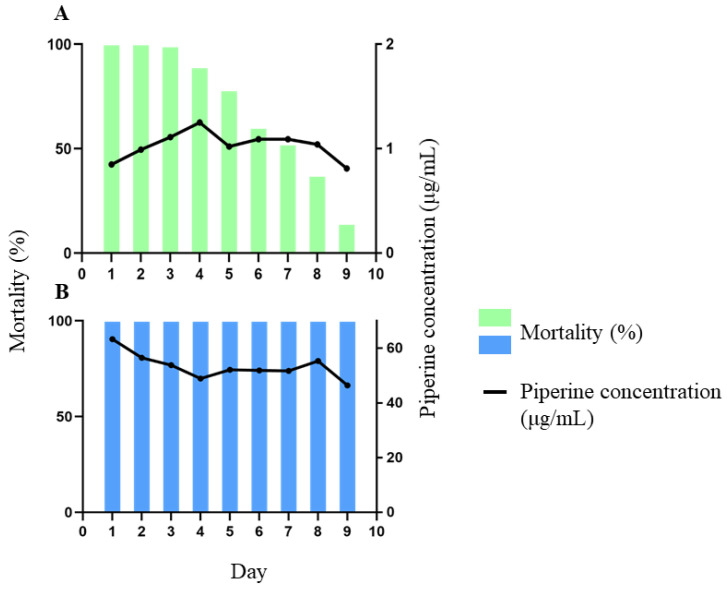
Residual larvicidal activity against *Aedes aegypti* (L3) and piperine quantification: (**A**). *Piper nigrum* standardized extract (green), initial concentration 4 μg/mL; (**B**). Piperine (blue), initial concentration 60 μg/mL.

After LC_50_/LC_90_ determination, 60 μg/mL piperine and 4 μg/mL extract were selected for residual larvicidal activity investigation (Section 3.8), and the piperine was quantified daily to understand the behavior of this major compound. However, piperine did not achieve adequate solubility at this test concentration, with precipitation observed. Therefore, the addition of the exhausted matrix to the test solution ensured accuracy by enabling piperine solubility. Another challenge was analytical error determination during piperine quantification in water. Three different piperine solutions were prepared in plastic cups: 47.3 µg/mL (error 37%), 23.6 µg/mL (error 41%) and 4.58 µg/mL (error 54%). The corresponding errors that were determined were applied as a correction factor in the piperine quantification calculation during the residual larvicidal assay. As the aforementioned quantification obstacles were overcome, the piperine and standardized extract assays were performed according to Section 3.8. The daily mortality (%) and absolute piperine concentration (µg/mL) results of the 9-day test are shown in Figure 5.

The extract achieved 100% mortality over the first three days, with piperine concentration between 0.85 and 1.11 μg/mL. Mortality reduced to 89% on day 4 (1.25 μg/mL), and 78% on day 5 (1.02 μg/mL), declining from 60 (day 6) and 52% (day 7) both with piperine 1.09 μg/mL to 37% on day 8 (1.04 μg/mL). On the final day, only 14% of larvae were dead (0.81 μg/mL) (Figure 5A). The average piperine concentration was 1.03 ± 0.13 μg/mL (RSD 12%). The accuracy study determined 17% maximum error during quantification. Given that the piperine concentration did not significantly reduce, the residual larvicidal activity observed herein, together with the LC_50_ values after 24 h exposure (extract 1.1 μg/mL and piperine 19.03 μg/mL), highlight the probable important role of minority compounds.

Figure 5B shows piperine caused 100% larvicidal activity over the 9-day experiment, with the average concentration 53.4 ± 4.6 μg/mL (RSD 8.7%), a variation previously observed during the accuracy test. The lowest piperine concentration was 48.8 μg/mL (day 9, 100% mortality), higher than the LC_90_ values (Table 3), confirming adequate lethal concentration calculations.

The LC_50_ and LC_90_ values suggest that larvae mortality is related to the combination of piperine concentration, and the minority compounds detected (Figure 4B,C). A previous study [26] correlating the quality control of *P. nigrum* samples and *Ae. aegypti* larvae did not include all the parameters validated herein. Furthermore, the quantification in the literature involved separated samples while the present study sampled from the same biological test.

### 2.6. Small-Scale Simulated Field Trial of P. nigrum Fruit Ethanolic Extract Solid Formulation against Aedes aegypti Larvae

**Figure 6:**
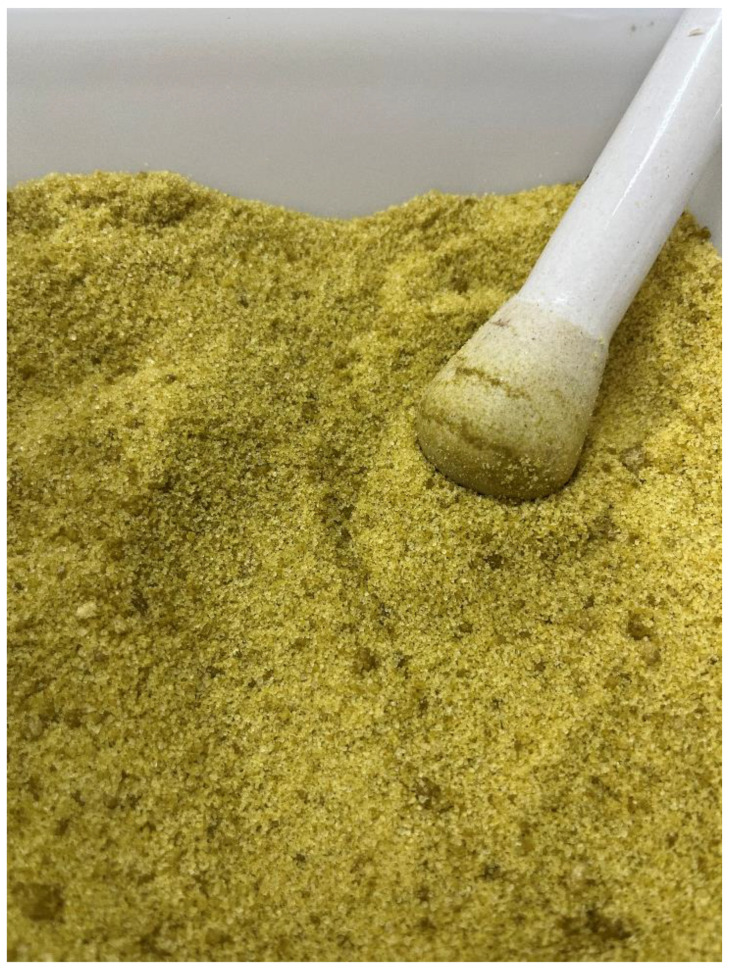
Solid formulation of *Piper nigrum* fruit ethanolic extracts produced by ASE-150/ultrasound assisted maceration (UAM) extraction.

In addition to the standardized *P. nigrum* fruit ethanolic extract, an ultrasound assisted maceration (UAM) extract was performed to increase extract yield, with both extracts subsequently added to granulated white sugar to facilitate application formulation, each containing 50 mg extract:1 g (Section 3.9, Figure 6).

**Figure 7:**
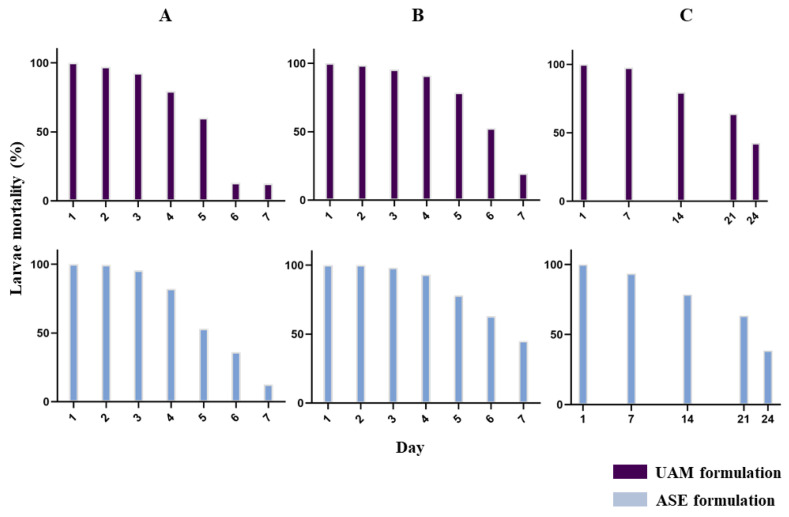
Small-scale simulated field trial of *Piper nigrum* fruit ethanolic standardized (ASE) and ultrasound assisted maceration (UAM) extract formulations (granulated white sugar) against *Aedes aegypti* L3 larvae. (**A**). 2.5 μg/mL. (**B**). 5 μg/mL. (**C**). 10 μg/mL.

Both formulations were tested in 10 L: 0.5 g (2.5 μg/mL), 1 g (5 μg/mL) and 2 g (10 μg/mL) (Figure 7).

Both formulations caused 100% mortality on day 1 at all three concentrations. The assay was halted on day 7 for the 2.5 and 5 μg/mL formulations, as the average mortality reached <50%. The 10 μg/mL assay, however, continued until day 24 when larvae mortality dropped below 50% for both the ASE and UAM formulations (Table S16). On comparison with the activity observed in the piperine quantification test (7 days, Figure 4) (Section 3.5), it was possible to observe that the activity of the extract was maintained in the formulation and that 10 μg/mL provided prolonged residual activity until day 24 (>3 fold). A simple rapid incorporation process, which is beneficial in terms of cost effectiveness, is deemed favorable, particularly due to prolonged activity at 10 μg/mL. No records were found in the literature involving field or semi-field trails of black pepper against *Ae. aegypti*. *Piper nigrum* is available all around the world, known as “The King of Spices”, and is obtainable in large quantities. In 2020, Brazil ranked second in *P. nigrum* production. In 2018, a total of 752,000 tons were produced globally [27]. Extract yield and insecticidal activity at lower concentrations means that 1 kg of powdered *P. nigrum* could treat 6000 L of water. All these data provide evidence that a product developed on this basis could contribute to treating a huge public health concern.

## 3. Materials and Methods

### 3.1. Instrumentation and HPLC Analysis

An ASE-150 (Accelerated Solvent Extraction, Thermo Fisher^®^, Sunnyvale, CA, USA) apparatus with 100 mL stainless steel cells was used for extractions. A Waters HPLC system, photodiode array detector 2998, autosampler 2707 and binary HPLC pump 1525 (Waters, Milford, MA, USA) were used for analysis and method validation. Chromatographic separation was performed using a Kinetex Biphenyl TMS end-capped column, 4.6 × 150 mm with 5 μm particle size (Phenomenex^®^, Torrance, CA, USA). The mobile phase contained 0.2% formic acid in water (phase A) or acetonitrile (phase B). The total run time was 21 min: gradient elution started with 40% phase B reaching 45% in 1 min, continued until 9 min when the phase reached 80%. Phase B was subsequently raised by 5% over 4 min (85%) and maintained for 3 min. After one more min, B reached 100%, before returning to the initial phase in 1 min, where it was maintained for 3 min. The 10 μL sample was injected at 1 mL/min.

### 3.2. Edible Plant Extractions

Plants were purchased at local markets in Brasilia, DF, Brazil. The material was powdered and passed through a 1.4 mm sieve (12 mesh) or chopped when fresh. After size reduction, the material was submitted to ASE-150^®^ extraction, at 70 °C, with 3 static 5 min cycles, 60% rinse volume, a 150-sec purge time using hexane:dichloromethane:ethyl acetate:ethanol (4:1:4:1). All of the matrices extracted are listed in Table 1.

### 3.3. Optimized Extraction

Following the general extractions, one of the matrices (*Piper nigrum* L.) was selected to optimize the extraction parameters using ASE-150^®^. To select the extraction solvent, 9 previous extracts prepared using: ethanol (50/90/130 °C), ethyl acetate:ethanol (1:9, 50/90/130 °C) and ethanol:water (9:1, 50/90/130 °C) were tested against *Ae. aegypti* L3 larvae. A Box-Behnken design was applied using 3 continued factors: cell sample quantity (1–5 g), extraction temperature (50–130 °C) and static cycle duration (2–4 min). Ethanol and 2 static cycles were fixed parameters. A total of 15 different extractions (in duplicate) were conducted varying: temperature, sample quantity and static cycle duration. The resulting extracts were tested at 2.5, 2.0 and 1.9 μg/mL against *Ae. aegypti* L3 larvae. The response yield (%) and larvae mortality at 1.9 μg/mL allowed optimized extraction selection. All data was analyzed using the Minitab^®^ 18 software (State College, PA, USA).

### 3.4. Sample Preparation

The optimized extraction sample was prepared using the ASE-150^®^ apparatus as follows: 1 g powdered black pepper was transferred to the extraction cell and extracted with ethanol at 50 °C, with two 4-min static cycles, 60% rinse volume and a 150-sec purge time. Of the resulting 175 mL solution obtained, 1 mL was filtered (0.22 μm × 13 mm nylon filter) and transferred to a glass vial for analysis. The ≥97% piperine standard solution, previously isolated (Figure S12) or purchased (Merck, Burlington, MA, USA), was analytically prepared and diluted with ethanol.

### 3.5. Method Validation

The method was validated using the parameters specified by the regulatory agencies. [28] Selectivity, linearity, limits of detection and quantification (LOD, LOQ), stability, precision, accuracy, matrix effect and robustness were assessed. Selectivity was obtained by chromatographic profile comparison between the piperine standard and the optimized extraction, retention time (RT), α factor, peak width and height were monitored. UV spectra of the standard and samples were observed during the experiments. Linear regression was plotted as peak area as a function of concentration. Regarding linearity, piperine curves were plotted from serially-dilutions (in triplicate): content (11.5; 23.1; 46.1; 92.3; 184.5, and 263.6 µg/mL) and impurity (1.5; 3.0; 6.1; 12.1; 24.3, and 48.5 µg/mL). Residue statistical analysis was also performed. LOD and LOQ were calculated from standard deviation of the y-intercept and the calibration curve slope, similar to linearity. For stability, piperine solutions and ASE-150-prepared extracts were submitted to variations in temperature and light over 3 days (9 h light/15 h dark) at 2–8 °C. After 24 h, the relative concentration was determined. Precision was determined by repeatability, with 6 extractions performed by one analyst on the same day, while intermediate precision was performed by 2 analysts on 2 consecutive days. The peak area, retention time (RT) and tail factor were monitored. Accuracy was determined by recovery, involving construction of a matrix by exhaustion (Soxhlet, 16 h, 9:1 ethanol:acetone) and drying. Fifteen 1 g matrix samples were grouped, with 5 samples at 3 different levels. Aliquots (1 mL) of piperine solution were added at 10, 20 and 40 mg/mL for low, medium and high level, respectively. After drying, these piperine-enriched matrices were submitted to ASE-150 extraction at the pre-determined conditions. Since the optimized extraction obtained 175 mL of solution (Section 3.4), the 3 respective theoretical concentrations the method had to reach were: 57.14, 114.28 and 228.57 µg/mL (Table S13). After extraction, samples were prepared for analysis (Section 2.4). Normalized matrix effect (NME) was calculated from the calibration curves constructed for piperine (163; 81; 41; 20, and 10 µg/mL) and the piperine-enriched *P. nigrum* extract (143; 138; 125; 99 and 48 µg/mL of piperine). Analysis was performed in triplicate. Piperine standard solution was proportionally added to the extract. The NME was calculated by the slope ratios between the 2 analytical curves. Robustness was determined using a complete factorial design 2^4^, with the variations listed in Table 2. Retention time and peak area were monitored in 16 experiments, in duplicate. The factor effect calculation was transformed at relative standard deviation (RSD) using the formula: RSD (%) = (S/X).100, in which S is the calculated effect value and X the mean y response, considering different responses and factors [29]. All parameters analyzed during the method evaluation were reported as RSD (%) values between the different measures. System adequacy was determined (Table S17) to ensure correct method execution.

### 3.6. Validated Method Applicability for Other Piper nigrum L. Samples

Method applicability was investigated by absolute piperine quantification in 10 different commercially acquired samples: 5 black pepper/5 white pepper. Samples were powdered, with subsequent extraction and sample preparation performed as in Section 3.4. Peak area, retention time and relative piperine content were determined for each sample.

### 3.7. Biological Assays

The *Aedes aegypti* (Rockefeller) strain used herein is maintained at the Laboratório de Farmacognosia Insectarium at the Universidade de Brasília under the following controlled conditions: 28 °C (±2), 70% (±10) relative humidity (RH) and a 12/12 h light/dark photoperiod. Egg hatching occurred under reduced pressure in tap water. Larvae were fed with fish food until pupae formation. The pupae were grouped into males and females (at a ratio of 1:3, respectively) and transferred to the cage. The newly emerged mosquitoes were fed with a 10% sugar solution, with equine blood (Hospital Veterinário of the Universidade de Brasília) available 3 times per week for egg production [1]. Screening of L3 instar larvae and pupae were conducted in a 12-well plate, with 3 mL and 10 individuals/well [1]. Extracts diluted in DMSO (dimethyl sulfoxide < 2%) were tested at 250 µg/mL, with DMSO used as the negative control. After the initial screening, *P. nigrum* was selected for extraction optimization. The different *P. nigrum* extracts optimized by ASE-150^®^ were submitted to larvae screening in 12-well plates as previously described (Section 3.3) at 1.9 µg/mL. The WHO protocol [30] was adopted for LC_50_ and LC_90_ determination. Briefly, 200 mL water was added to transparent plastic cups, each containing 25 L3 larvae. The extract was subsequently added at the respective concentrations (2.5; 1.5; 1.1; 0.9 and 0.5 µg/mL). Piperine was tested at 50; 25; 15; 10 and 5 µg/mL. Three independent tests were performed in quadruplicate. Larvae mortality was evaluated after 24, 48 and 72 h. DMSO (1%) and temephos (6.25; 3.13; 1.76; 0.58 and 0.39 ng/mL) were used as the negative and positive controls, respectively.

### 3.8. Piperine Quantification: Laboratory Larvicidal Assays

The piperine standard and standardized *P. nigrum* fruit ethanolic extract were tested against L3 larvae: (i) an aqueous solution containing 500 µg/mL constructed matrix (Section 3.5), 0.5% ethanol and piperine (at 60 µg/mL, approximately 3-fold the LC_50_ value respecting the CI 95%), and (ii) an aqueous solution containing 0.5% ethanol and extract (at 4 µg/mL, approximately 2-fold LC_90_ value respecting the CI 95%). These solutions were added to transparent plastic cups, (200 mL, in quadruplicate) each containing 25 larvae, and were weighed. The negative control was performed with matrix in water and ethanol or ethanol only at the concentrations used. Every 24 h, larvae mortality was recorded, viable larvae were removed, 25 other were larvae added, water evaporation was monitored by measuring the weight of the plastic cups and were adjusted if necessary. For each extract, a total of 20 mL test solution was transferred to a glass vial, 5 mL from each of the 4 replicates, in order to maintain sufficient solution column for larvae movement, and the cup was reweighed to enable evaporation determination the next day. The 20 mL vial sample was frozen, lyophilized, resuspended in 1 mL ethanol and prepared as previously described (Section 3.4) for HPLC analysis. For the piperine samples, 1 mL test solution from each cup was transferred to an individual vial and 1 mL ethanol added. After dilution and homogenization, the piperine samples were prepared as described in Section 3.4.

### 3.9. Small-Scale Simulated Field Trial of P. nigrum Fruit Ethanolic Extracts in Formulation: Standardized and UAM

For the formulation, two *P. nigrum* extraction methods were employed: optimized ASE-150 (Section 3.4) and ultrasound-assisted maceration (UAM). The latter was performed in two 30-min periods with 500 g powdered *P. nigrum* fruit in 1 L ethanol, with the solvent renewed between periods. The extractive solution was dried by rotary evaporation. Both the ASE-150 and maceration (UAM) extracts were individually diluted in ethanol (10 mg/mL) and were added to crystallized white sugar (10 mL:2 g), transferred to a Petri dish and then completely dried overnight. The resulting solid preparations (Figure 6) were tested in buckets containing 10 L water and 100 *Ae. aegypti* L3 larvae, in 3 quantities (2, 1 and 0.5 g) corresponding to 10, 5 and 2.5 µg/mL extract, respectively (concentrations >LC_90_ recommended in the WHO protocol) [30]. Tests were performed in duplicate. Daily mortality was recorded, with viable larvae removed and replaced (100 larvae/bucket).

### 3.10. Statistical Analysis

LC_50_ and LC_90_ values were determined using nonlinear regression with 4 parameters, together with one-way ANOVA and Dunnett’s post-test to compare different groups using the GraphPad Prism 9 software (GraphPad Software, San Diego, CA, USA). *p* values of < 0.05 were considered significant. Microsoft Excel^®^ was used for residue analysis and basic statistics.

## 4. Conclusions

The present study screened edible plant extracts for activity against *Aedes aegypti* larvae and pupae. *Piper nigrum* was selected to produce a standardized optimized extract validated in accordance with WHO protocols and the rigorous parameters required by regulatory agencies. In addition, piperine was quantified directly from test samples with piperine only and with standardized extract, in the same method. Produced using a green solvent, this ASE-150^®^ extract can be similarly produced by ultrasound assisted maceration (UAM) to increase yield with similar activity. Formulations of both extracts in granulated white sugar proved effective in small-scale simulated field trials.

This cost-effective rapid incorporation process resulted in a 10 μg/mL formulation with prolonged activity of up to 24 days. This investigation overcomes obstacles limiting the use of natural products for arboviral vector control. The use of a validated crude extract obtained from a highly consumed widely available spice, formulated in such a simple way with prolonged residual activity against *Aedes aegypti* larvae at low concentrations, constitutes a viable opportunity to address this significant public health concern.

## Figures and Tables

**Table 1:** Larvicidal/pupicidal activity (250 µg/mL, 24 h) and yield of extracts produced by accelerated solvent extraction (ASE-150).

Family	Nº	Common Name	Species	Plant	Larvae	Pupae	Yield
				Part	Mortality (%)	Mortality (%)	(%)
Alliaceae	1	onion	*Allium cepa* L.	Bulb	*-*	*-*	1
	2	purple onion	*Allium cepa* L.	Bulb	*-*	*-*	1.3
Alismataceae	3	Florida burrhead	*Echinodorus grandiflorus* Micheli.	AP	100	70	2.6
Amaranthaceae	4	amaranth	*Amaranthus caudatus* L.	Se	36	*-*	2.1
	5	quinoa	*Chenopodium quinoa* Willd.	Se	6	*-*	1.7
Annonaceae	6	sugar apple	*Annona squamosa* L.	F (pulp)	26	*-*	0.2
	7	sugar apple	*Annona squamosa* L.	F (peel)	*-*	*-*	0.2
Arecaceae	8	coconut	*Cocos nucifera* L.	F	*-*	*-*	62.6
Asteraceae	9	chamomile	*Matricaria chamomilla* L.	Fl	100	26	4.4
	10	artichoke	*Cynara scolymus* L.	L	*-*	5	4.7
	11	carqueja	*Baccharis trimera* (Less.) DC.	AP	*-*	*-*	7.1
	12	marigold	*Calendula officinalis* L.	Fl	*-*	23	9
	13	carqueja	*Baccharis gaudichaudiana* DC.	AP	*-*	6	7.8
	14	sunflower	*Helianthus annus* L.	Se	6	3	41
	15	yacon	*Smallanthus sonchifolius* (Poepp.) H. Rob.	S	*-*	100	0.3
Brassicaceae	16	mustard	*Brassica alba* (L.) Rabenh.	Se	*-*	43	0.6
Cactaceae	17	pitaya	*Hylocereus monacanthus* (Lem.) Britton & Rose.	F (pulp)	*-*	*-*	2
	18	pitaya	*Hylocereus monacanthus* (Lem.) Britton & Rose.	F (peel)	100	*-*	0.05
Cucurbitaceae	19	maroon cucumber	*Cucumis anguria* L.	F	*-*	6	0.4
	20	pumpkin	*Cucurbita* sp.	Se	*-*	*-*	32.7
Celastraceae	21	*Espinheira santa*	*Maytenus ilicifolia* Mart. ex Reissek.	AP	*-*	*-*	2.1
Equisetaceae	22	bottlebrush	*Equisetum arvense* L.	S	10	30	2.1
Ericaceae	23	blueberry (dried)	*Vaccinium myrtillus* L.	F	100	33	0.7
	24	blueberry (fresh)	*Vaccinium myrtillus* L.	F	*-*	*-*	2.1
	25	cranberry	*Vaccinium macrocarpon* Aiton.	F	*-*	10	1.0
Fabaceae	26	mulungu	*Erythrina verna* Vell.	SW	33	95	0.3
	27	*Olho-de-boi*	*Dioclea violacea* Mart. ex Benth.	Se	*-*	100	0.3
	28	tamarind	*Tamarindus indica* L.	Se	*-*	66	0.7
	29	common beans	*Phaseolus vulgaris* L.	F	*-*	*-*	0.5
	30	pea	*Pisum sativum* L.	F	*-*	*-*	0.9
Humiriaceae	31	yellow uxi	*Endopleura uchi* (Huber) Cuatrec.	S	*-*	6	0.9
Lamiaceae	32	chia	*Salvia hispanica* L.	Se	50	5	1.8
Lauraceae	33	avocado	*Persea americana* Mill.	F (peel)	100	12	0.5
	34	avocado	*Persea americana* Mill.	F (pulp)	100	100	0.2
	35	avocado	*Persea americana* Mill.	Se	6	*-*	2.7
	36	hass avocado	*Persea americana* Mill.	F (pulp)	*-*	36	0.5
	37	hass avocado	*Persea americana* Mill.	Se	100	6	1.9
	38	hass avocado	*Persea americana* Mill.	F (peel)	12	6	0.6
	39	sassafras	*Sassafras albidum* (Nutt.) Nees.	S	100	10	0.2
Linaceae	40	golden linseed	*Linum usitatissimum* L.	Se	*-*	*-*	25.4
Lythraceae	41	pomegranate	*Punica granatum* L.	F (peel)	*-*	*-*	0.7
	42	pomegranate	*Punica granatum* L.	Se	*-*	*-*	3.2
Malvaceae	43	cocoa	*Theobroma cacao* L.	Sep	6	100	7.6
	44	cocoa	*Theobroma cacao* L.	Se	*-*	*-*	22.7
Melastomataceae	45	*canela de velho*	*Miconia albicans* (Sw) Steud.	AP	*-*	50	2.1
Moraceae	46	white mulberry	*Morus alba* L.	AP	*-*	5	3.2
	47	carapiá	*Dorstenia brasiliensis* Lam.	RW	67	3	0.6
Moringaceae	48	moringa	*Moringa oleifera* Lam.	L	26	*-*	7.9
Pedaliaceae	49	white sesame	*Sesamum indicum* L.	Se	*-*	*-*	3.6
Piperaceae	50	monkey pepper	*Piper aduncum* L.	Fl	47	*-*	3
	51	black pepper	*Piper nigrum* L.	F	100	*-*	6
Poaceae	52	lemon grass	*Cymbopogon citratus* (DC) Stapf	L	20	*-*	2.7
Ranunculaceae	53	black sesame	*Nigella sativa* L.	Se	*-*	*-*	45.1
Rutaceae	54	Sicilian lemon	*Citrus limon* (L.) Osbeck	F (peel)	56	6	0.3
	55	Sicilian lemon	*Citrus limon* (L.) Osbeck	F (pulp)	*-*	*-*	1.8
Solanaceae	56	goji berry	*Lycium barbarum* L.	F	100	36	1.7
	57	rosemary	*Rosmarinus officinalis* L.	L	*-*	*-*	12.6
	58	chili pepper	*Capsicum* sp.	F	100	100	0.4
	59	chocolate pepper	*Capsicum* sp.	F	23	12	1.9
	60	yellow scorpion pepper	*Capsicum* sp.	F	100	94	0.3
	61	red scorpion pepper	*Capsicum* sp.	F	7	46	0.07
	62	smelling pepper	*Capsicum* sp.	F	10	6	0.9
	63	cumari pepper	*Capsicum* sp.	F	100	23	0.3
	64	pepper goat	*Capsicum* sp.	F	*-*	3	0.8
	65	lady finger pepper	*Capsicum* sp.	F	*-*	6	1.6
	66	jurubeba	*Solanum paniculatum* L.	F	*-*	*-*	0.3
Verbenaceae	67	bushy matgrass	*Lippia alba* (Mill) N.E.Br.	L	2.5	25	3.3
Vitaceae	68	white raisin	*Vitis vinifera* L.	F	100	6	0.5
Zingiberaceae	69	cardamom	*Elettaria cardamomum* (L.) Maton	Sep	100	56	3.6
	70	ginger	*Zingiber officinale* Roscoe	R	*-*	*-*	0.5

AP: aerial parts; Se: seed; F: fruit; Fl: flower; L: leaves; S: stem; SW: stem wood; Sep: powdered seed; RW: root wood; R: root. -: inactive, no mortality observed. Solvent used: hexane:dichloromethane:ethyl acetate:ethanol (4:1:4:1). Nº: sample number.

**Table 2:** Variations made to determine method robustness.

Level	Factor			
	T (min)	FM	*λ_nm_*	Flow (mL.min^−1^)
−1	3	PVDF	256	0.9
+1	5	PTFE	276	1.1
Conventional method	4	nylon	266	1.0

T: ASE-150 extraction time static cycle; FM: filter membrane used for sample preparation; PVDF: polyvinylidene difluoride; PTFE: polytetrafluoroethylene; λnm: wavelength (nm) used in the UV-PDA detector.

**Table 3:** Larvicidal activity of piperine and standardized *P. nigrum* ethanolic fruit extract against *Ae. aegypti*.

Sample	Concentration (µg/mL)	LC_50_ µg/mL (CI 95%)	LC_90_ µg/mL (CI 95%)	R^2^ 24 h
piperine	50 25 15 10 5	19.03 (17.6–20.7) * 11.3 (10.2–12.4) ** 7.5 (6.2–8.8) ***	38.1 (31.8–45.4) * 27.6 (22.1–34. 4) ** 23.8 (17.2–33.3) ***	0.87
standardized *P. nigrum* ethanolic fruit extract	2.5 1.5 1.1 0.9 0.5	1.1 (1.1–1.2) * 0.9 (0.6–0.9) ** 0.9 (0.8–1.0) ***	1.8 (1.6–1.9) * 1.3 (1.2–1.5) ** 1.2 (1.0–1.4) ***	0.92
temephos	0.00625 0.00313 0.00156 0.00078 0.00039	0.0011 (0.0010–0.0012) * 0.0010 (0.0010–0.0011) ** 0.0009 (0.0009–0.0010) ***	0.00165 (0.00154–0.00175) * 0.00156 (0.00141–0.00170) ** 0.00129 (0.0011–0.00146) ***	0.98

LC_50_: lethal concentration, 50% individuals; LC_90_: lethal concentration, 90% individuals; CI: confidence interval. Data expressed as the average of 3 independent experiments in quadruplicate, total of 1500 larvae per sample. Temephos: positive control. R2: correlation coefficient. *: 24 h, **: 48 h, ***: 72 h.

## Data Availability

Not applicable.

## Supplementary Materials

The following supporting information can be downloaded at: https://www.mdpi.com/article/10.3390/molecules28031264/s1, Figure S1: Analytical curve of piperine at content level; Figure S2: Analytical curve of piperine at impurity level; Figure S3: Residue plot for linear regression at content level for piperine. A. Residue of estimated Y; B. Standard residue (%); Figure S4: Residue plot for linear regression at impurity level for piperine. A. Residue of estimated Y; B. Standard residue (%); Figure S5: Chromatographic profile of piperine at 0.81 µg/mL with a retention time of 6.9 min; Figure S6: Stability considering absolute piperine content over 72 h; Figure S7: Stability considering relative content of 13 studied peaks of *Piper nigrum* fruit ethanolic extract; Figure S8: Linear regressions for matrix effect determination. PS: Piperine standard; PS + SE: Piperine standard + Standardized extract of *P. nigrum*; (PS + SE) − IPC: Standardized extract enriched with piperine standardized solution minus initial piperine concentration at standardized extract; Figure S9: Plot for robustness data with RSD (Relative standard deviation) for piperine in the standardized extract. A) Robustness for retention time; B) Robustness for peak area; Figure S10: Dose-response curve for piperine against *Ae. aegypti* L3 larvae; Figure S11: Dose-response curve for standardized *P. nigrum* fruit ethanolic extract against *Ae. aegypti* L3 larvae; Figure S12: ^1^H NMR spectrum (600 MHz, CDCl3) of isolated piperine; Table S1: Box–Behnken experimental design for *P. nigrum* fruits, including the studied variables, response yields and mortality of *Aedes aegypti* L3 larvae at 1.9 μg/mL. ASE-150^®^ using ethanol; Table S2: Selectivity: chromatographic aspects evaluated for each peak (*n*= 6) of the standardized extraction; Table S3: Chromatographic variables evaluated for analytical curve of content level in triplicate, for piperine; Table S4: Chromatographic variables evaluated for analytical curve at impurity level in triplicate for piperine; Table S5: Residue results described for regression at content level of piperine; Table S6: Residue results described for regression at impurity level for piperine; Table S7: Analysis of variance (ANOVA) of regression: piperine at content level; Table S8: Analysis of variance (ANOVA) of the regression: piperine at impurity level; Table S9: Evaluated stability parameters for piperine standard solution (*n* = 3); Table S10: Evaluated stability parameters for *P. nigrum* fruit ethanolic extract (*n* = 3); Table S11: Repeatability data: *P. nigrum* fruit ethanolic extract (*n* = 6); Table S12: Intermediate precision data: *P. nigrum* fruit ethanolic extract (*n* = 6);Table S13: Data for determination of piperine method accuracy; Table S14: Normalized matrix effect (NME) data for piperine following the method; Table S15: Method applicability in different *P. nigrum* fruit samples; Table S16: Mortality of *Ae. aegypti* L3 larvae exposed to formulations of *P. nigrum* fruit ethanolic extracts at small-scale field trial; Table S17: Evaluation of system adequacy, ratio between two standard solutions (P1 and P2) for method development. Error < 3%.

## Sample Availability

Piperine sample is available from the authors.
