# Supplementary material for: Edible Plant Extracts against Aedes aegypti and Validation of a Piper nigrum L. Ethanolic Extract as a Natural Insecticide
Source: Molecules. 2023 Jan 28;28(3):1264. doi: 10.3390/molecules28031264 (PMC9921162; doi:10.3390/molecules28031264)
Supplement: Supplementary file 1 [file molecules-28-01264-s001.zip › molecules-2136632-supplementary.pdf]

Edible plant extracts against *Aedes aegypti* and validation of a *Piper nigrum* L. ethanolic extract as a natural insecticide

Lais Silva Morais[1], João Paulo Barreto Sousa[1], Carolina Melo Aguiar[1], Ciro Martins Gomes[2], Daniel Pecoraro Demarque[1,3], Lorena Carneiro Albernaz[1] and Laila Salmen Espindola[1*]

[1] Laboratório de Farmacognosia, Universidade de Brasília, Campus Universitário Darcy Ribeiro, Asa Norte, Brasília, CEP 70910-900, DF, Brazil

[2] Programa de Pós-Graduação em Ciências Médicas, Faculdade de Medicina, Universidade de Brasília, Campus Universitário Darcy Ribeiro, Asa Norte, Brasília, CEP 70910-900, DF, Brazil

[3] Laboratório de Farmacognosia, Faculdade de Ciências Farmacêuticas, Universidade de São Paulo, Av. Professor Lineu Prestes, 580, São Paulo, CEP 05508-900, SP, Brazil

* Corresponding author: darvenne@unb.br (L. S. Espindola)

Abstract: The *Aedes aegypti* mosquito significantly impacts public health, with vector control remaining the most efficient means of reducing the number of arboviral disease cases. This study screened the larvicidal and pupicidal activity of common edible plant extracts. *Piper nigrum* L. (black pepper) extract production was optimized using accelerated solvent extraction (ASE) and validated following regulatory requirements using HPLC-PDA analytical methodology to quantify its major component – piperine. Larvicidal activity was determined for the standardized *P. nigrum* fruit ethanol extract (LC_{50} 1.1 μg/mL) and piperine standard (LC_{50} 19.0 μg/mL). Furthermore, 9-day residual activity was determined for the extract (4 μg/mL) and piperine (60 μg/mL), with daily piperine quantification. Semi-field trials of solid extract formulations demonstrated 24-day activity against *Ae. aegypti* larvae. Thus, the standardized *P. nigrum* extract emerges as a potential candidate for insecticide development to control the arboviral vector.

Keywords: *Aedes aegypti*; *Piper nigrum* L.; black pepper; accelerated solvent extraction (ASE); standardized extract; validation

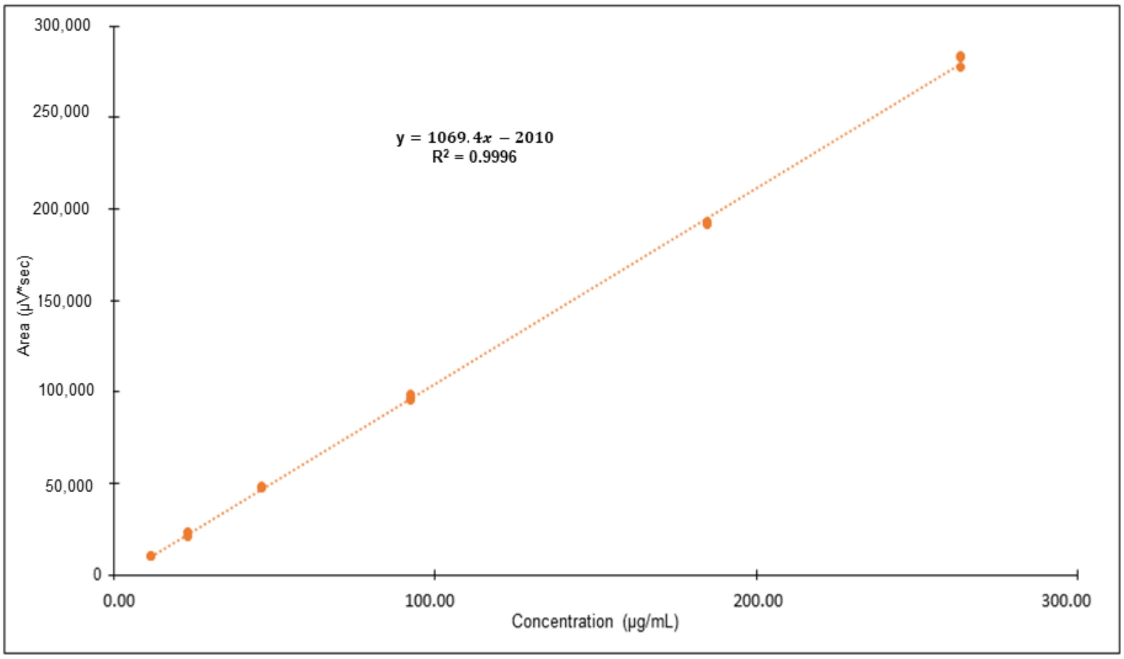

Figure S1. Analytical curve of piperine at content level.

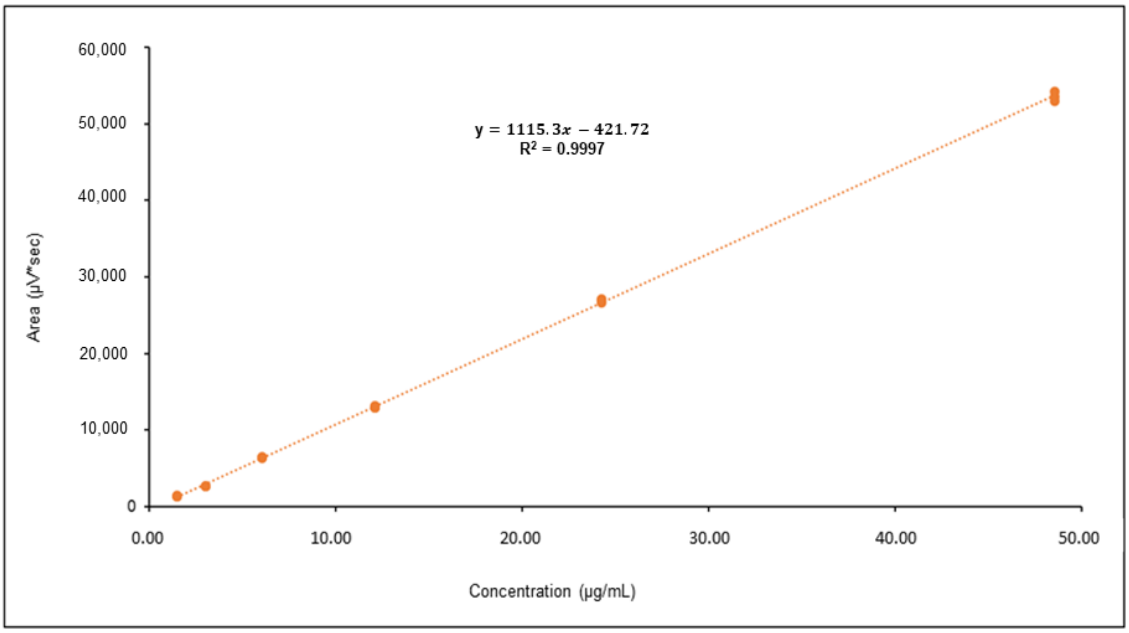

Figure S2. Analytical curve of piperine at impurity level.

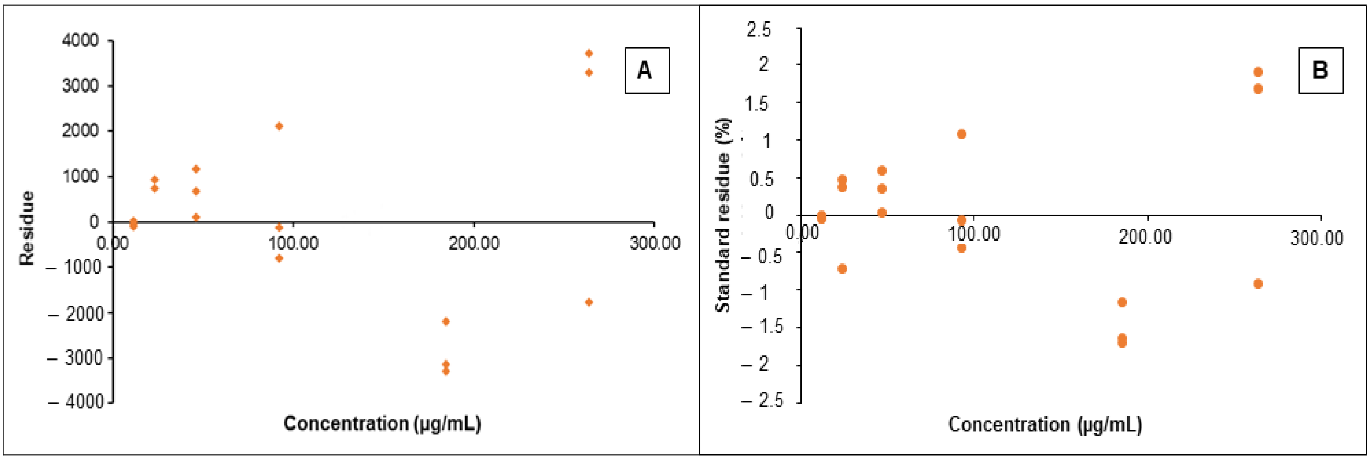

Figure S3. Residue plot for linear regression at content level for piperine. **A.** Residue of estimated Y; **B.** Standard residue (%).

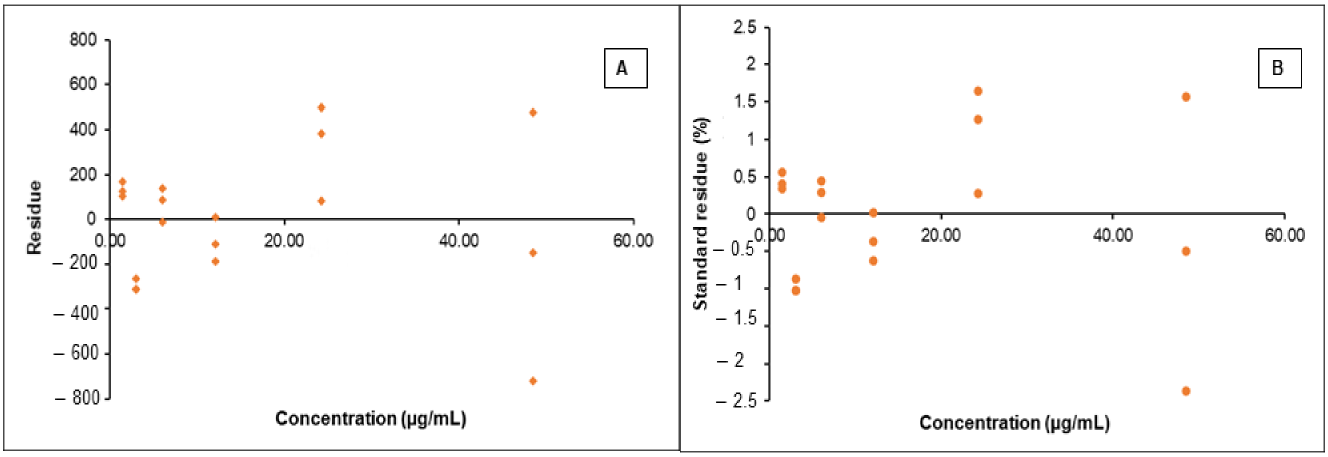

Figure S4. Residue plot for linear regression at impurity level for piperine. **A.** Residue of estimated Y; **B.** Standard residue (%).

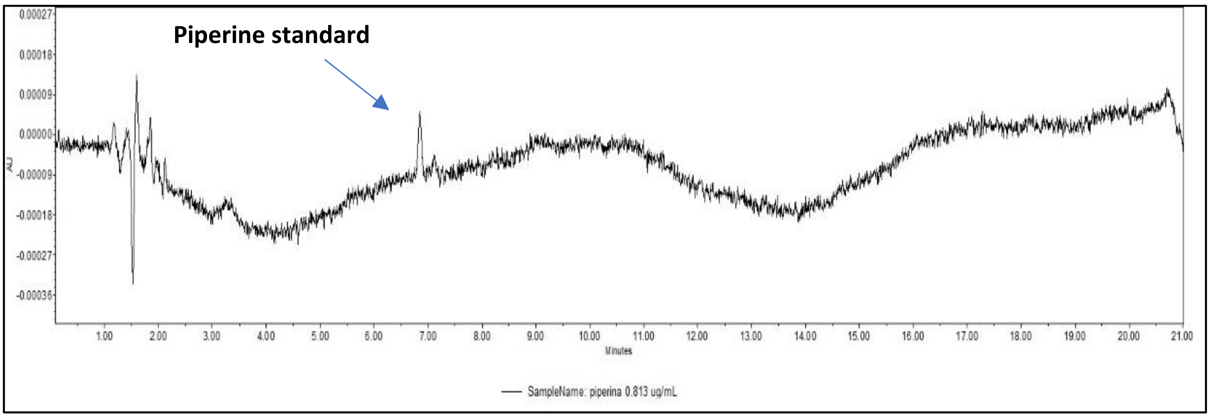

Figure S5. Chromatographic profile of piperine at 0.81 μg/mL with a retention time of 6.9 min.

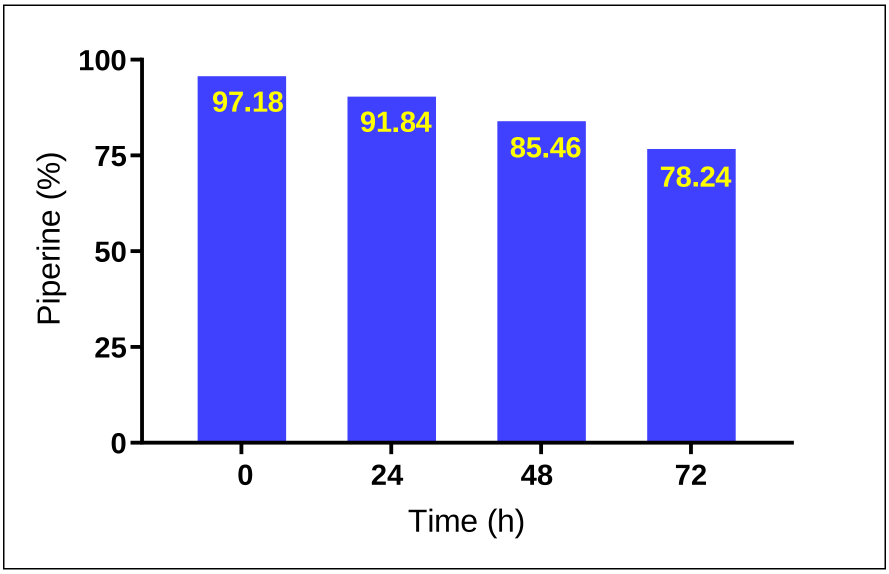

Figure S6. Stability considering absolute piperine content over 72 h.

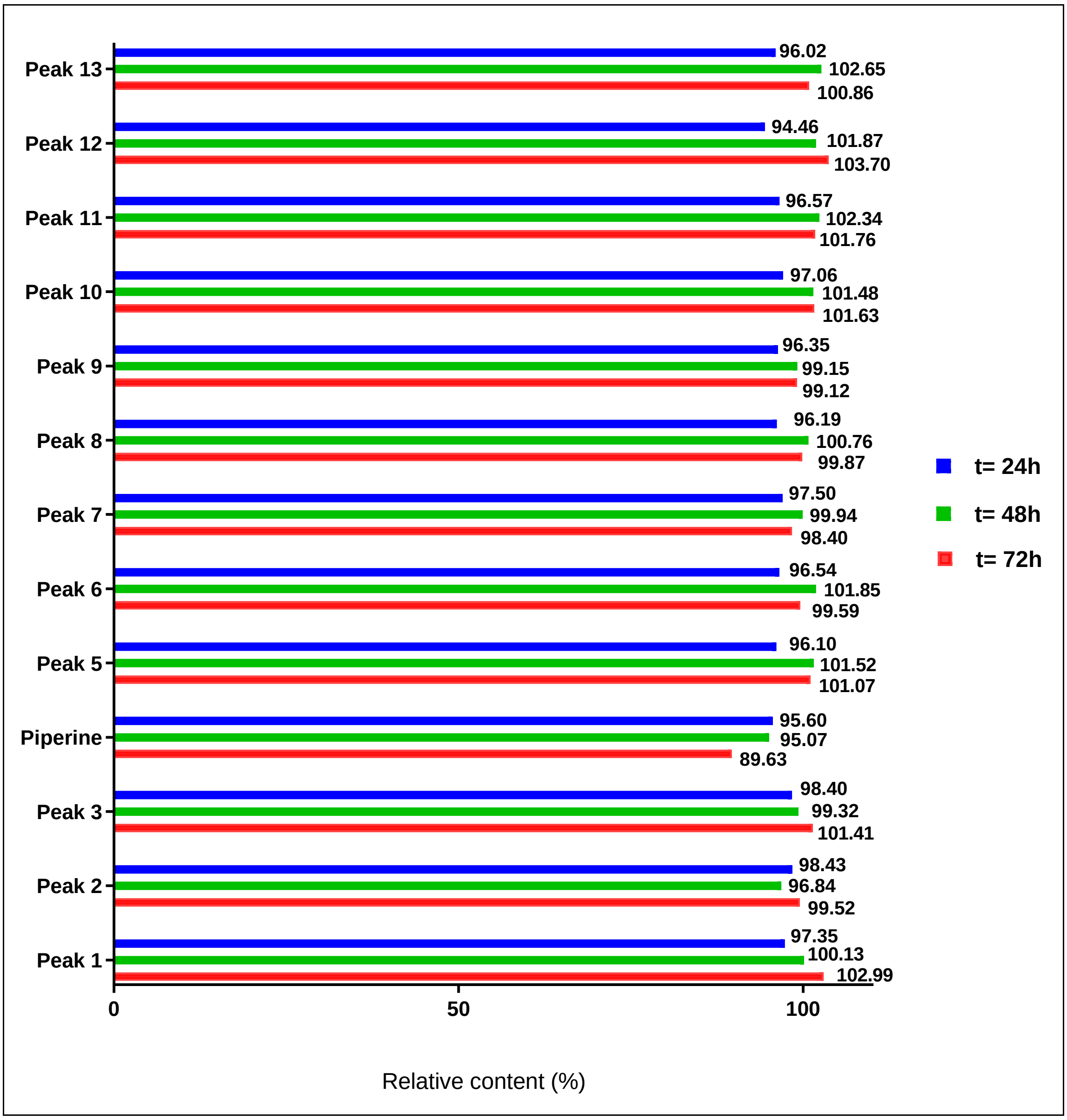

Figure S7. Stability considering relative content of 13 studied peaks of *Piper nigrum* fruit ethanolic extract.

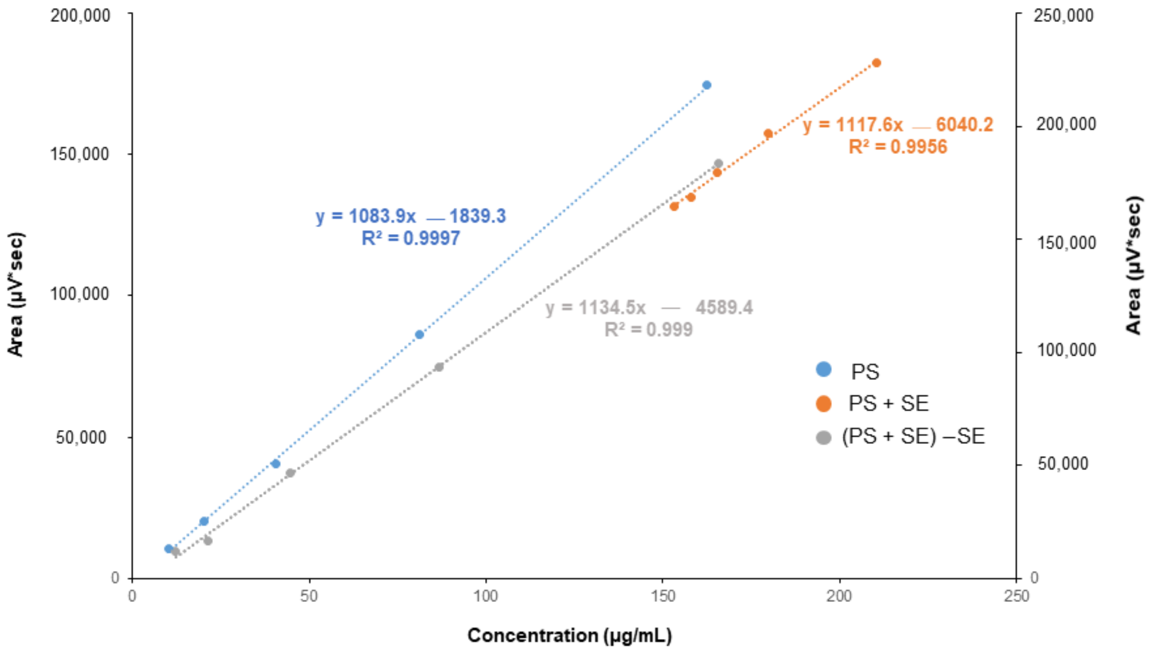

Figure S8. Linear regressions for matrix effect determination. PS: Piperine standard; PS+ SE: Piperine standard + Standardized extract of *P. nigrum*; (PS+SE) – IPC: Standardized extract enriched with piperine standardized solution minus initial piperine concentration at standardized extract.

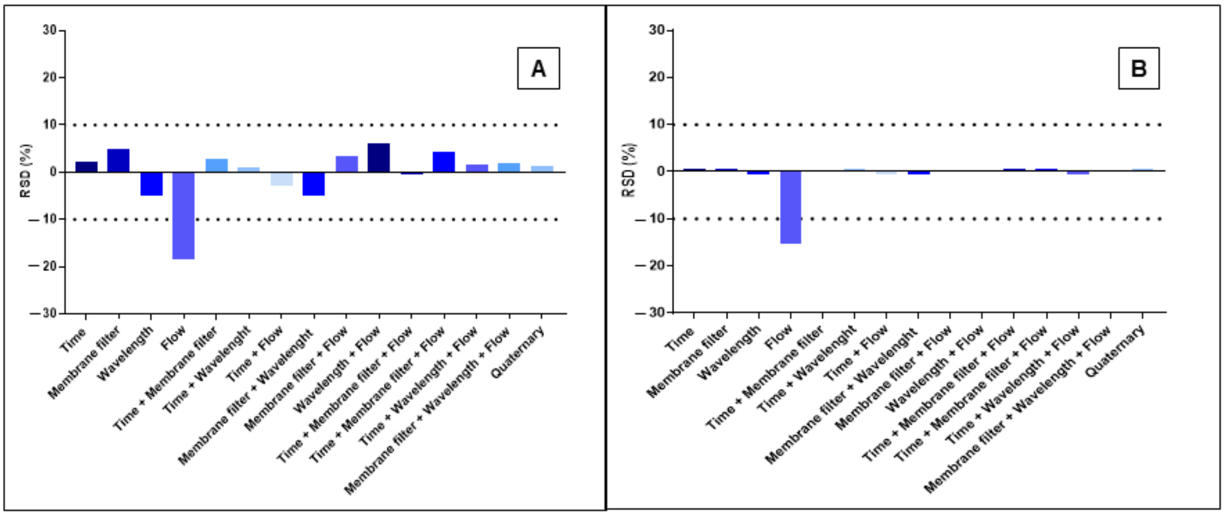

Figure S9. Plot for robustness data with RSD (Relative standard deviation) for piperine in the standardized extract. **A)** Robustness for retention time; **B)** Robustness for peak area.

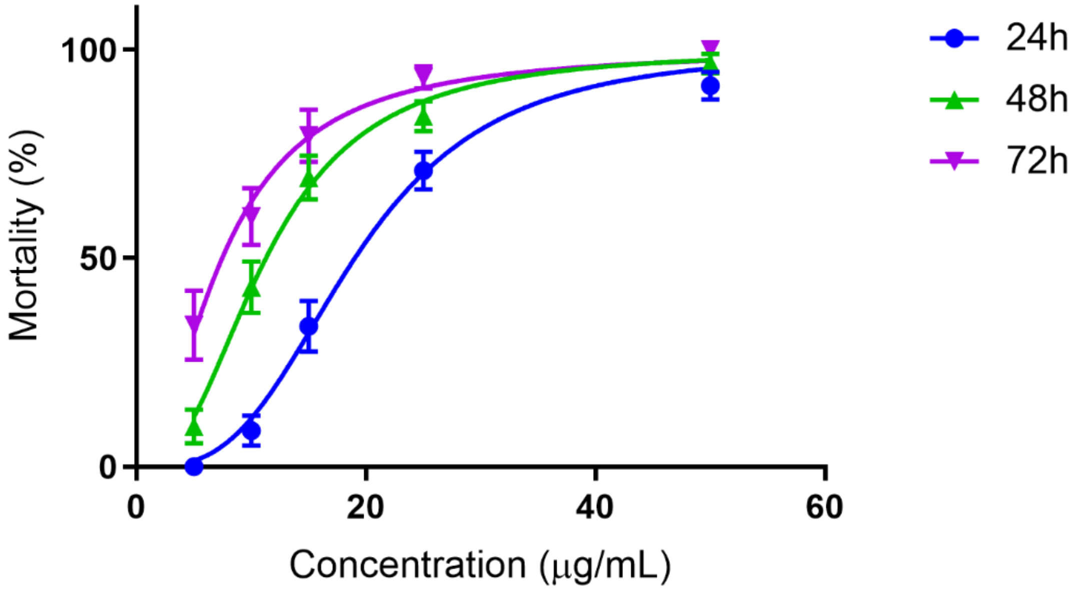

Figure S10. Dose-response curve for piperine against *Ae. aegypti* L3 larvae.

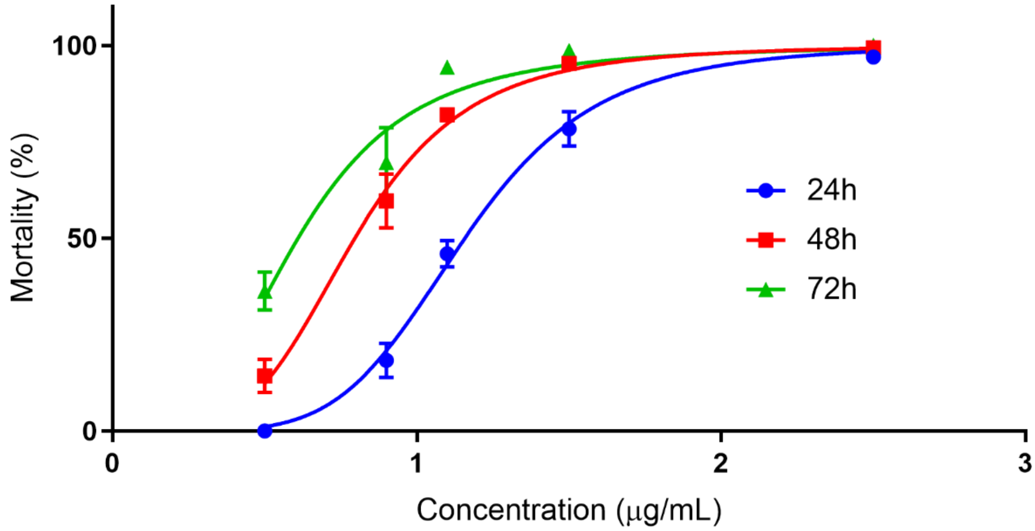

Figure S1. Dose-response curve for standardized *P. nigrum* fruit ethanolic extract against *Ae. aegypti* L3 larvae.

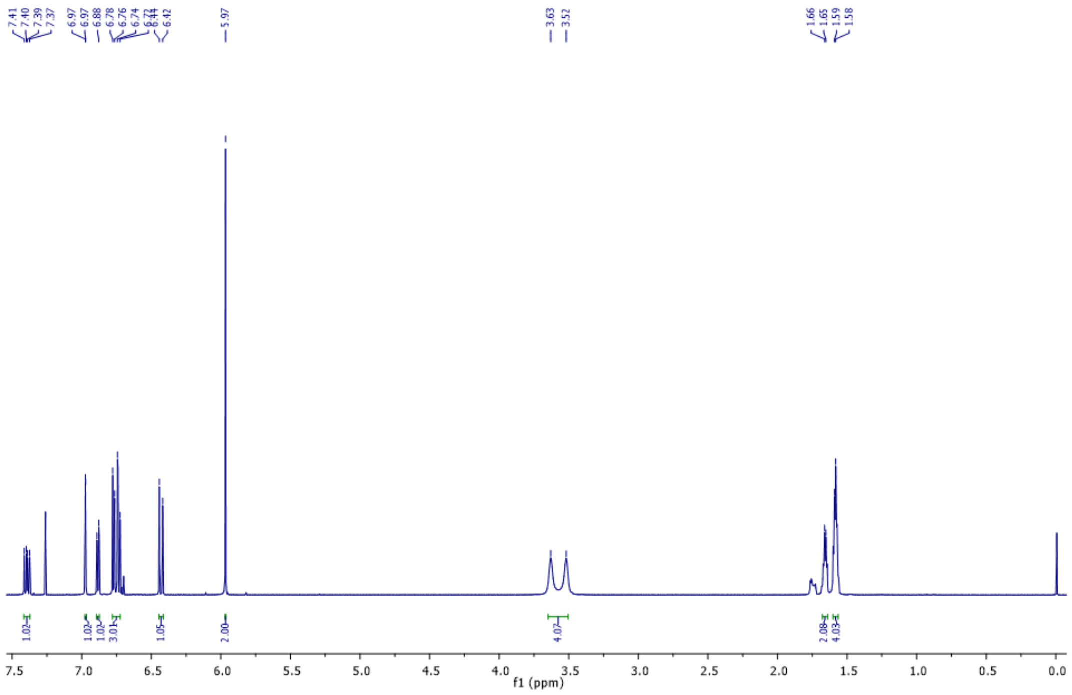

Figure S22. ^{1}H NMR spectrum (600 MHz, CDCl3) of isolated piperine.

Table S1. Box-Behnken experimental design for *P. nigrum* fruits, including the studied variables, response yields and mortality of *Aedes aegypti* L3 larvae at 1.9 μg/mL. ASE-150® using ethanol.

Experiment	Time (min.)	Temperature (ºC)	Sample (g)	Mortality (%)	Yield (%)
A	2	130	3	30.8	9.1
B	3	130	1	61.7	9.7
C	4	50	3	84.2	6.5
D	4	90	1	74.2	7.1
E	4	90	5	78.3	7.9
F	4	50	3	86.7	6.8
G	2	50	3	80.8	4.9
H	3	130	1	78.3	9.5
I	3	130	5	23.3	10.1
J	3	90	3	60.8	8.9
K	2	90	5	53.3	11
L	3	90	3	88.3	7.4
M	3	90	3	75	7
N	2	90	5	86.7	6.9
O	3	50	1	92.5	6.4
P	2	90	1	71.7	7.7
Q	2	50	3	90	6.1
R	3	90	3	76.7	7
S	3	50	5	80.8	6.2
T	4	90	1	70	6.8
U	2	90	1	52.5	7.1
V	4	130	3	68.3	8.3
W	2	130	3	60	9.1
X	4	90	5	65	10.8
Y	4	130	3	19.2	13
Z	3	50	5	82.5	6.6
AA	3	130	5	67.5	8.5
AB	3	90	3	61.7	7.1
AC	3	90	3	43.3	10
AD	4	50	1	94.2	5.9

Table S2. Selectivity: chromatographic aspects evaluated for each peak (n= 6) of the standardized extraction.

N°	RT (min) ±SD	RSD (%)	Fα ± SD	RSD (%)	PH ± SD	RSD (%)	PW ± SD	RSD (%)
PS	6.87 ± 0.005	0.07	1.04 ± 0.001	0.07	33280 ± 216	0.65	14.2 ± 0.17	1.22
1	5.91 ± 0.015	0.25	1.05 ± 0.0008	0.08	1173 ± 36	3.06	10.4 ± 0.40	3.88
2	6.22 ± 0.014	0.23	1.06 ± 0.0008	0.08	600 ± 18	2.93	10.6 ± 0.41	3.92
3	6.61 ± 0.015	0.22	1.05 ± 0.0003	0.03	773 ± 30	3.82	9.3 ± 0.29	3.10
4	6.93 ± 0.015	0.22	1.09 ± 0.0003	0.03	35781 ± 847	2.37	14.3 ± 0.20	1.42
5	7.58 ± 0.015	0.20	1.06 ± 0.0002	0.02	4408 ± 131	2.96	13.0 ± 0.48	3.71
6	8.04 ± 0.016	0.19	1.06 ± 0.0003	0.03	1200 ± 35	2.95	11.1 ± 0.48	4.35
7	8.53 ± 0.016	0.18	1.10 ± 0.0004	0.03	4051 ± 193	4.76	10.5 ± 0.41	3.86
8	9.36 ± 0.016	0.16	1.04 ± 0.0002	0.02	2229 ± 76	3.43	9.4 ± 0.25	2.68
9	9.78 ± 0.016	0.17	1.11 ± 0.0004	0.03	4902 ± 169	3.45	10.9 ± 0.39	3.60
10	10.88 ± 0.015	0.14	1.10 ± 0.0002	0.02	3471 ± 98	2.83	10.7 ± 0.50	4.72
11	11.99 ± 0.017	0.14	1.06 ± 0.0006	0.06	3822 ± 129	3.37	16.5 ± 0.50	3.04
12	12.75 ± 0.019	0.15	1.03 ± 0.0004	0.04	1467 ± 54	3.66	13.9 ± 0.35	2.48
13	13.15 ± 0.019	0.14	1.10 ± 0.0010	0.09	2514 ± 101	4.01	17.3 ± 0.70	4.04

N°: peak number; PS: piperine standard; RT: retention time; Fα: factor of separation; PH: peak height; PW: peak width (sec); SD: standard deviation; RSD: relative standard deviation (%).

Table S3. Chromatographic variables evaluated for analytical curve of content level in triplicate, for piperine.

Level	Curve	RT (min)	Area	PH
1 (263.6 μg/mL)	1	6.88	283617	61605
	2	6.87	278136	60985
	3	6.89	283183	61454
	mean	6.88	281645	61348
	SD	0.010	3047	323
	RSD (%)	0.14	1.08	0.53
2 (184.5 μg/mL)	1	6.89	192047	42429
	2	6.89	192184	42570
	3	6.89	193128	42705
	mean	6.89	192453	42568
	SD	0.003	589	138
	RSD (%)	0.04	0.31	0.32
3 (92.3 μg/mL)	1	6.91	98766	21108
	2	6.90	95855	20861
	3	6.90	96544	20976
	mean	6.90	97055	20982
	SD	0.002	1521	124
	RSD (%)	0.03	1.57	0.59
4 (46.1 μg/mL)	1	6.91	48503	10441
	2	6.90	48012	10387
	3	6.90	47422	10318
	mean	6.90	47979	10382
	SD	0.003	541	62
	RSD (%)	0.05	1.13	0.59
5 (23.1 μg/mL)	1	6.90	23590	5122
	2	6.91	23395	5097
	3	6.92	21304	4699
	mean	6.91	22763	4973
	SD	0.004	1267	237
	RSD (%)	0.06	5.57	4.77
6 (11.5 μg/mL)	1	6.90	10242	2266
	2	6.91	10339	2274
	3	6.91	10281	2234
	mean	6.91	10287	2258
	SD	0.002	49	21
	RSD (%)	0.03	0.47	0.94

RT: retention time; PH: peak height; SD: standard deviation; RSD: relative standard deviation (%).

Table S4. Chromatographic variables evaluated for analytical curve at impurity level in triplicate for piperine.

Level	Curve	RT (min)	Area	PH
1 (48.5 μg/mL)	1	6.90	54167	11949
	2	6.95	52974	11785
	3	6.92	53541	11773
	mean	6.92	53561	11836
	SD	0.027	597	98
	RSD (%)	0.39	1.11	0.83
2 (24.3 μg/mL)	1	6.91	26719	5925
	2	6.90	27134	5939
	3	6.90	27018	5899
	mean	6.90	26957	5921
	SD	0.006	214	20
	RSD (%)	0.09	0.79	0.34
3 (12.1 μg/mL)	1	6.90	12917	2852
	2	6.91	12994	2833
	3	6.91	13114	2828
	mean	6.91	13008	2838
	SD	0.003	99	13
	RSD (%)	0.04	0.76	0.45
4 (6.1 μg/mL)	1	6.91	6330	1394
	2	6.91	6431	1377
	3	6.91	6479	1391
	mean	6.91	6413	1387
	SD	0.003	76	9
	RSD (%)	0.04	1.19	0.65
5 (3.0 μg/mL)	1	6.92	2697	595
	2	6.92	2648	593
	3	6.91	2649	620
	mean	6.92	2665	603
	SD	0.002	28	15
	RSD (%)	0.03	1.05	2.5
6 (1.5 μg/mL)	1	6.90	1439	330
	2	6.91	1372	310
	3	6.92	1393	317
	mean	6.91	1401	319
	SD	0.008	34	10
	RSD (%)	0.11	2.45	3.18

RT: retention time; PH: peak height; SD: standard deviation; RSD: relative standard deviation (%).

Table S5. Residue results described for regression at content level of piperine.

OBS.	Conc. (µg/mL)	Experimental Y	Estimated Y	Res.	S.Res .	Residual Rec. (%)	Curve Rec. (%)
1	263.6	283617	279897	3720	1.93	98.69	101.32
2	263.6	278136	279897	-1761	-0.91	100.63	99.38
3	263.6	283183	279897	3286	1.70	98.84	101.17
4	184.5	192047	195325	-3278	-1.70	101.71	98.34
5	184.5	192184	195325	-3141	-1.63	101.63	98.41
6	184.5	193128	195325	-2197	-1.14	101.14	98.89
7	92.3	98766	96657	2109	1.09	97.86	102.14
8	92.3	95855	96657	-802	-0.42	100.84	99.19
9	92.3	96544	96657	-113	-0.06	100.12	99.89
10	46.1	48503	47324	1179	0.61	97.57	102.40
11	46.1	48012	47324	688	0.36	98.57	101.40
12	46.1	47422	47324	98	0.05	99.79	100.20
13	23.1	23590	22657	933	0.48	96.04	103.79
14	23.1	23395	22657	738	0.38	96.84	103.00
15	23.1	21304	22657	-1353	-0.70	106.35	94.52
16	11.5	10242	10323	-81	-0.04	100.79	99.34
17	11.5	10339	10323	16	0.01	99.85	100.13
18	11.5	10281	10323	-42	-0.02	100.41	99.66

OBS.: observations; Conc: concentration; Res: residue; S. Res.: standard residue; Residual Rec.: residual recovery, relation of experimental data and regression in relation of y axis; Curve Rec.: recovery of the regression expressed by X axes.

Table S6. Residue results described for regression at impurity level for piperine.

OBS.	Conc. (μg/mL)	Experimental Y	Estimated Y	Res.	S.Res .	Residual Rec. (%)	Curve Rec. (%)
1	48.5	54167	53692	475	1.57	99.12	100.88
2	48.5	52974	53692	-718	-2.37	101.35	98.67
3	48.5	53541	53692	-151	-0.5	100.28	99.72
4	24.3	26719	26635	84	0.28	99.69	100.31
5	24.3	27134	26635	499	1.65	98.16	101.84
6	24.3	27018	26635	383	1.26	98.58	101.41
7	12.1	12917	13107	-190	-0.63	101.47	98.6
8	12.1	12994	13107	-113	-0.37	100.87	99.17
9	12.1	13114	13107	7	0.02	99.94	100.05
10	6.1	6330	6342	-12	-0.04	100.2	99.81
11	6.1	6431	6342	89	0.29	98.62	101.31
12	6.1	6479	6342	137	0.45	97.89	102.02
13	3	2697	2960	-263	-0.87	109.77	92.21
14	3	2648	2960	-312	-1.03	111.8	90.76
15	3	2649	2960	-311	-1.03	111.75	90.79
16	1.5	1439	1269	170	0.56	88.21	110.03
17	1.5	1372	1269	103	0.34	92.52	106.07
18	1.5	1393	1269	124	0.41	91.12	107.31

OBS.: observations; Conc: concentration; Res: residue; S. Res.: standard residue; Residual Rec.: residual recovery, relationship of experimental data and regression in relation to the *y* axis; Curve Rec.: recovery of the regression expressed by the *x* axis.

Table S7. Analysis of variance (ANOVA) of regression: piperine at content level.

	ANOVA					
	DF	SS	MS	F	Fs	Ft
regression	1	1.73E+11	1.73E+11	43761	6.25392E-29	4.49
residue	16	6.34E+07	3.96E+06			
Total	17	1.73E+11				
	Coefficient	SD	Stat t	*P*-value	95% lower	95% upper
intersection	-2010.01	707.26	-2.84	0.0118	-3509.34	-510.68
inclination	1069.45	5.11	209.19	6.2539E-29	1058.61	1080.29

DF: degrees of freedom; SS: sum of squares; MS: mean of squares; F: coefficient of *Fisher* test for analysis of the differences between regression and residue; Fs: P- value of F test; Ft: F- table; SD: standard deviation; Stat t: student t test for significance of coefficients; *P*-value <0.05; 95% lower: lower limit of 95% confidence interval; 95% upper: upper limit of 95% confidence interval.

Table S8. Analysis of variance (ANOVA) of the regression: piperine at impurity level.

ANOVA						
	DF	SS	MS	F	Fs	Ft
regression	1	6035233961	6035233961	61886.5	3.91E-30	4.49
residue	16	1560335.9	97521			
Total	17	6036794297				
	Coefficient	SD	Stat *t*	*P*- value	95% Low.	95% Upp.
intersction	-421.72	102.53	-4.11	0.001	-639.07	-204.37
inclination	1115.28	4.48	248.77	3.90E-30	1105.78	1124.78

DF: degrees of freedom; SS: sum of squares; MS: mean of squares; F: coefficient of *Fisher* test for analysis of the differences between regression and residue; Fs: P- value of F test; Ft: F- table; SD: standard deviation; Stat t: student t test for significance of coefficients; *P*-value: <0.05; 95% lower: lower limit of 95% confidence interval; 95% upper: upper limit of 95% confidence interval.

Table S9. Evaluated stability parameters for piperine standard solution (n = 3).

T (h)	RT ± SD	RSD (%)	Peak area ± SD	RSD (%)
0	6.88 ± 0.004	0.06	168182 ± 546	0.33
24	6.87 ± 0.001	0.02	158914 ± 1707	1.07
48	6.86 ± 0.004	0.05	147883 ± 1356	0.92
72	6.87 ± 0.002	0.03	135387 ± 382	0.28

RT: retention time (min); SD: standard deviation; RSD: relative standard deviation; T: exposure time (h).

Table S10. Evaluated stability parameters for *P. nigrum* fruit ethanolic extract (n = 3).

N°	Time (h)	RT ± SD	RSD (%)	Peak area ± SD	RSD (%)
1	0	5.85 ± 0.003	0.05	4896 ± 99	2.03
	24	5.85 ± 0.006	0.11	4767 ± 129	2.7
	48	5.86 ± 0.005	0.08	4903 ± 113	2.31
	72	5.85 ± 0.003	0.06	5043 ± 159	3.16
2	0	6.16 ± 0.005	0.09	2813 ± 113	4.02
	24	6.16 ± 0.008	0.12	2769 ± 94	3.4
	48	6.17 ± 0.003	0.05	2724 ± 109	4.01
	72	6.16 ± 0.003	0.05	2800 ± 105	3.76
3	0	6.55 ± 0.003	0.05	3074 ± 60	1.95
	24	6.55 ± 0.009	0.14	3025 ± 85	2.8
	48	6.56 ± 0.002	0.03	3053 ± 150	4.93
	72	6.55 ± 0.002	0.04	3117 ± 100	3.21
4	0	6.86 ± 0.003	0.05	168859 ± 1219	0.72
	24	6.86 ± 0.005	0.07	161430 ± 1687	1.04
	48	6.88 ± 0.003	0.05	160535 ± 886	0.55
	72	6.87 ± 0.003	0.04	151349 ± 1178	0.78
5	0	7.52 ± 0.002	0.03	18467 ± 109	0.59
	24	7.52 ± 0.002	0.08	17747 ± 217	1.22
	48	7.52 ± 0.003	0.03	18747 ± 241	1.29
	72	7.52 ± 0.003	0.03	18664 ± 198	1.06
6	0	7.99 ± 0.003	0.04	4564 ± 31	0.68
	24	7.98 ± 0.005	0.06	4406 ± 87	1.97
	48	7.99 ± 0.002	0.03	4648 ± 49	1.05
	72	7.98 ± 0.002	0.03	4545 ± 98	2.15
7	0	8.48 ± 0.003	0.03	20184 ± 413	2.04
	24	8.48 ± 0.005	0.06	19588 ± 289	1.48
	48	8.48 ± 0.005	0.06	20171 ± 190	0.94
	72	8.47 ± 0.003	0.03	19861 ± 311	1.57
8	0	9.31 ± 0.004	0.04	8418 ± 183	2.18
	24	9.31 ± 0.004	0.05	8097 ± 219	2.7
	48	9.31 ± 0.006	0.07	8481 ± 88	1.04
	72	9.30 ± 0.003	0.03	8407 ± 228	2.71
9	0	9.72 ± 0.004	0.04	21940 ± 278	1.27
	24	9.72 ± 0.004	0.04	21139 ± 262	1.24
	48	9.72 ± 0.006	0.06	21754 ± 149	0.68
	72	9.72 ± 0.003	0.03	21748 ± 759	3.49
10	0	10.83 ± 0.002	0.02	13730 ± 180	1.31
	24	10.82 ± 0.006	0.06	13327 ± 184	1.38
	48	10.83 ± 0.006	0.05	13934 ± 48	0.35
	72	10.82 ± 0.001	0.01	13955 ± 221	1.58
11	0	11.93 ± 0.001	0.01	22916 ± 420	1.83
	24	11.92 ± 0.006	0.05	22129 ± 405	1.83

	48	11.93 ± 0.007	0.06	23453 ± 355	1.52
	72	11.92 ± 0.002	0.02	23319 ± 361	1.55
12	0	12.69 ± 0.006	0.05	10213 ± 421	4.12
	24	12.68 ± 0.006	0.05	9647 ± 214	2.22
	48	12.69 ± 0.007	0.05	10403 ± 142	1.37
	72	12.68 ± 0.003	0.02	10591 ± 522	4.93
13	0	13.08 ± 0.007	0.06	19214 ± 329	1.71
	24	13.07 ± 0.003	0.02	18449 ± 431	2.34
	48	13.09 ± 0.008	0.06	19722 ± 332	1.69
	72	13.07 ± 0.005	0.04	19379 ± 340	1.75

N°: peak number; RT: retention time; SD: standard deviation; RSD: relative standard deviation.

Table S11. Repeatability data: *P. nigrum* fruit ethanolic extract (n=6).

N°	RT ± SD	RSD (%)	Peak area ± DP	RSD (%)	TF ± SD	RSD (%)
1	5.90 ± 0.004	0.07	5430 ± 199	3.67	0.12 ± 0.0028	2.39
2	6.21 ± 0.004	0.06	3167 ± 89	2.82	0.07 ± 0.0016	2.31
3	6.60 ± 0.005	0.08	3402 ± 95	2.79	0.11 ± 0.0028	2.64
4	6.91 ± 0.005	0.07	169034 ± 4279	2.53	5.75 ± 0.1401	2.43
5	7.55 ± 0.006	0.08	20417 ± 803	3.93	0.93 ± 0.0340	3.66
6	8.02 ± 0.005	0.06	4979 ± 137	2.76	0.30 ± 0.0063	2.11
7	8.51 ± 0.004	0.05	18700 ± 838	4.48	1.24 ± 0.0677	5.47
8	9.34 ± 0.004	0.04	9326 ± 347	3.72	0.88 ± 0.0266	3.01
9	9.75 ± 0.004	0.04	24481 ± 895	3.65	2.28 ± 0.0881	3.65
10	10.85 ± 0.004	0.03	15553 ± 680	4.37	2.21 ± 0.0881	3.99
11	11.95 ± 0.003	0.03	26559 ± 1067	4.02	3.27 ± 0.1481	4.52
12	12.69 ± 0.005	0.04	12062 ± 194	1.61	1.55 ± 0.0359	2.31
13	13.08 ± 0.003	0.02	22277 ± 945	4.24	2.86 ± 0.1174	4.10

N°: Peak number; RT: Retention time (min); TF: Tail factor; SD: Standard deviation; RSD: Relative standard deviation.

Table S12. Intermediate precision data: *P. nigrum* fruit ethanolic extract (n=6).

N°	RT ± SD	RSD (%)	Peak area ± DP	RSD (%)	TF ± SD	RSD (%)
1	5.90 ± 0.005	0.08	5357 ± 248	4.64	0.11 ± 0.005	4.16
2	6.21 ± 0.005	0.09	3121 ± 136	4.34	0.07 ± 0.004	5.70
3	6.59 ± 0.005	0.08	3336 ± 164	4.92	0.10 ± 0.004	3.85
4	6.91 ± 0.005	0.07	1690554 ± 6782	4.27	5.46 ± 0.217	4.02
5	7.56 ± 0.005	0.07	18912 ± 937	4.98	0.88 ± 0.044	5.04
6	8.03 ± 0.005	0.07	4786 ± 219	4.61	0.29 ± 0.013	4.59
7	8.52 ± 0.005	0.06	19562 ± 882	4.52	1.30 ± 0.078	6.06
8	9.34 ± 0.005	0.05	8861 ± 426	4.85	0.85 ± 0.043	5.13
9	9.76 ± 0.005	0.05	22858 ± 1112	4.86	2.16 ± 0.116	5.39
10	10.85 ± 0.005	0.05	14425 ± 696	4.85	2.09 ± 0.117	5.61
11	11.95 ± 0.004	0.03	25800 ± 760	2.93	3.14 ± 0.185	5.95
12	12.70 ± 0.006	0.05	11904 ± 298	2.51	1.50 ± 0.072	4.87
13	13.09 ± 0.005	0.04	22148 ± 779	3.51	2.77 ± 0.164	5.97

N°: Peak number; RT: Retention time (min); TF: Tail factor; SD: Standard deviation; RSD: Relative standard deviation.

Table S13. Data for determination of piperine method accuracy.

L	TC (µg/mL)	EC (µg/mL)	Concentration E (µg/mL)	E (%)	Rec (%)	RecM (%) ± SD	RSD (%)
High	228.57	221.1	7.47	3.27	96.73	92.73 ± 4.73	5.1
		202.35	26.22	11.47	88.53		
		203.37	25.2	11.03	88.97		
		204.41	24.16	10.57	89.43		
		228.59	-0.02	-0.01	100.01		
Medium	114.28	98.8	15.48	13.55	86.45	88.88 ± 4.23	4.76
		98.65	15.63	13.68	86.32		
		95.78	18.5	16.19	83.81		
		108.11	6.17	5.4	94.6		
		106.51	7.77	6.8	93.2		
Low	57.14	45.81	11.33	19.83	80.17	83.36 ± 4.75	5.7
		44.66	12.48	21.84	78.16		
		52.59	4.55	7.96	92.04		
		47.29	9.85	17.24	82.76		
		47.8	9.34	16.35	83.65		

L: Level of concentration; TC: Theorical concentration; EC: Experimental concentration; E: Error; Rec: Recovery; M: Mean; SD: Standard deviation; RSD: Relative standard deviation.

Table S14. Normalized matrix effect (NME) data for piperine following the method.

Curve	Regression	r^2	LDR (µg/mL)	P	MED			
PS	y = 1083.9x - 1839.3	0.9997	10.15 - 162.15	5	M ± SD AC	RSD AC %	NME	E%
PS+SE	y = 1117.6x - 6040.2	0.9956	153.02 - 210.56	5	1100.75 ± 24	2.16	0.97	± 3.02
(PS+SE)-IPC	y = 1134.5x - 4589.4	0.9990	12.12 - 165.70	5	1109.2 ± 36	3.23	0.96	± 4.46

PS: Piperine standard; PS+ SE: Piperine standard + Standardized extract of *Piper nigrum*; (PS+SE) – IPC: Standardized extract enriched with piperine standardized solution minus Initial piperine concentration present in the standardized extract. LDR: Linear dynamic range; P: Number of curve points; M: Mean; SD: Standard deviation; AC: Angular coeficient; RSD: Relative standard deviation; NME: Normalized matrix effect; E: Error

Table S15. Method applicability in different *P. nigrum* fruit samples.

Provider	Batch number	RT ± SD	RSD	AREA ± SD	RSD	Content %
RP	PNB-31102020	6.914 ± 0.011	0.165	124125 ± 8004	6.45	2.0
RC	PNB-OO8R	6.912 ± 0.004	0.052	115555 ± 3777	3.27	2.0
TC	PNB-14921	6.909 ± 0.006	0.086	96426 ± 2696	2.8	1.6
CF	PNB-70926	6.902 ± 0.019	0.276	96046 ± 6863	7.15	1.6
CTFG	PNB-30102020	6.894 ± 0.001	0.021	115941 ± 3533	3.05	1.9
CDQ	PNW-341	6.899 ± 0.004	0.064	141792 ± 6411	4.52	2.3
RP	PNW-30102020	6.908 ± 0.003	0.04	99920 ± 1134	1.13	1.7
TC	PNW-14921	6.925 ± 0.022	0.325	127618 ± 2119	1.66	2.1
CTFG	PNW-31102020	6.932 ± 0.002	0.032	130894 ± 9520	7.27	2.2
BM	PNW-32102020	6.927 ± 0.005	0.072	152313 ± 6150	4.04	2.5

RP: *Rei das Pimentas*; RC: *Rei dos condimentos*; TC: *Temperos e Cia*; CF: *Carrefour*; CTFG: *Casa de temperos Feira do Guará*; CDQ: *Casa de Doces e queijos*; BM: *Biomundo*; PNP: *Piper nigrum* black; PNW: *Piper nigrum* white; RT: Retention time; SD: Standard deviation; RSD: Relative standard deviation.

Table S16. Mortality of *Ae. aegypti* L3 larvae exposed to formulations of *P. nigrum* fruit ethanolic extracts at small-scale field trial.

	Mortality (mean, %)											
	2.5 μg/mL				5 μg/mL				10 μg/mL			
	ASE		UAM		ASE		UAM		ASE		UAM	
Day	Mean	SD	Mean	SD	Mean	SD	Mean	SD	Mean	SD	Mean	SD
1	100	0.0	100	0.0	100.0	0.0	100.0	0.0	100.0	0.0	100.0	0.0
2	99.5	0.7	97	1.4	100.0	0.0	98.5	0.7	100.0	0.0	99.5	0.7
3	95.5	2.1	92.5	7.8	97.0	1.4	95.5	0.7	99.0	1.4	99.5	0.7
4	82	2.8	79.5	2.1	94.0	1.4	91.0	1.4	98.5	0.7	98.0	1.4
5	53	15.6	60	21.2	88.0	14.1	78.5	9.2	95.5	0.7	97.0	1.4
6	36	15.6	13	11.3	67.5	6.4	52.5	30.4	96.0	2.8	91.5	0.7
7	12.5	12.0	12.5	9.2	52.5	10.6	19.5	4.9	93.5	4.9	97.5	3.5
8	-	-	-	-	-	-	-	-	94.5	0.7	83.5	3.5
9	-	-	-	-	-	-	-	-	82.5	6.4	83.5	3.5
10	-	-	-	-	-	-	-	-	85.5	4.9	61.0	8.5
11	-	-	-	-	-	-	-	-	82.5	3.5	71.0	9.9
12	-	-	-	-	-	-	-	-	82.0	5.7	80.5	0.7
13	-	-	-	-	-	-	-	-	80.0	0.0	80.5	2.1
14	-	-	-	-	-	-	-	-	78.5	9.2	79.5	2.1
15	-	-	-	-	-	-	-	-	83.0	11.3	86.5	0.7
16	-	-	-	-	-	-	-	-	92.5	6.4	96.5	0.7
17	-	-	-	-	-	-	-	-	79.5	4.9	83.0	8.5
18	-	-	-	-	-	-	-	-	90.0	8.5	86.5	7.8
19	-	-	-	-	-	-	-	-	86.5	3.5	82.5	6.4
20	-	-	-	-	-	-	-	-	85.0	2.8	82.0	11.3
21	-	-	-	-	-	-	-	-	63.5	0.7	64.0	1.4
22	-	-	-	-	-	-	-	-	65.0	1.4	58.5	16.3
23	-	-	-	-	-	-	-	-	62.5	3.5	67.5	2.1
24	-	-	-	-	-	-	-	-	44.0	5.7	42.5	2.1

UAM: Ultrasound assisted maceration formulation. ASE: ASE standardized formulation. - test halted on Day-7.

Table S17. Evaluation of system adequacy, ratio between two standard solutions (P1 and P2) for method development. Error <3 %.

P1				P2				Ratio %
Sample analysis	RT (min)	Area	TF	Sample analysis	RT (min)	Area	TF	
1	6.85	163340	10.69	1	6.86	163804	10.66	98.74
2	6.85	160543	10.55	2	6.85	163722	10.59	
3	6.85	160456	10.50	–	–	–	–	
4	6.86	162050	10.49	–	–	–	–	
5	6.86	160388	10.49	–	–	–	–	
6	6.85	162740	10.53	–	–	–	–	
mean	6.85	161586	10.54	mean	6.86	163763	10.62	
SD	0.01	1298	0.08	SD	0.01	58	0.05	
RSD (%)	0.08	0.80	0.73	RSD (%)	0.10	0.035	0.49	
Mass (mg)	15.03			Mass (mg)	15.04			
Content	0.9718			Content	0.9718			
Dilution	100			Dilution	100			
[C] μg/mL	146			[C] μg/mL	146			

RT: retention time; TF: tail factor; P1 and P2: piperine standard solutions; SD: standard deviation; RSD: relative standard deviation; [C]: concentration
